# Preclinical evaluation of a multi-epitope mRNA vaccine platform for broad and durable SARS-CoV-2 protection

**DOI:** 10.3389/fimmu.2026.1787877

**Published:** 2026-05-05

**Authors:** Laura Marcos-Villar, Beatriz Perdiguero, Laura Sin, Enrique Álvarez, Sara Flores, José M. Casasnovas, Tirso Pons, Carlos Oscar S. Sorzano, Daniel del Hoyo, Philipp Lapuhs, María J. Alonso, Mariano Esteban, Carmen Elena Gómez

**Affiliations:** 1 Department of Molecular and Cellular Biology, Centro Nacional de Biotecnología (CNB), Consejo Superior de Investigaciones Científicas (CSIC), Madrid, Spain; 2 Centro de Investigación Biomédica en Red de Enfermedades Infecciosas (CIBERINFEC), Instituto de Salud Carlos III (ISCIII), Madrid, Spain; 3 Department of Microbial Biotechnology, CNB, CSIC, Madrid, Spain; 4 Department of Immunology and Oncology, CNB, CSIC, Madrid, Spain; 5 Biocomputing Unit and Computational Genomics, CNB, CSIC, Madrid, Spain; 6 Department of Pharmacology, Pharmacy and Pharmaceutical Technology, Center for Research in Molecular Medicine and Chronic Diseases (CiMUS), Universidad de Santiago de Compostela, Santiago de Compostela, Spain; 7 IDIS Research Institute, Santiago de Compostela, Spain

**Keywords:** broad immunity, cross-protection, mRNA-LNP, multi-epitope vaccine, SARS-CoV-2

## Abstract

**Introduction:**

The emergence of immune-evasive SARS-CoV-2 variants has exposed limitations in the breadth and durability of protection conferred by current Spike-based vaccines, highlighting the need for next-generation approaches targeting conserved viral regions. Here, we describe the design and preclinical evaluation of an optimized multi-epitope vaccine, CoV2-BMEPu.

**Methods:**

CoV2-BMEPu was rationally designed using immunological data from SARS-CoV-2 convalescent cohorts, incorporating conserved and immunodominant regions from the Spike (S), Membrane (M) and Nucleocapsid (N) proteins, together with selected receptor-binding domain (RBD) segments associated with broadly neutralizing antibodies. The construct was engineered as a secreted trimeric antigen and delivered as an mRNA vaccine formulated in lipid nanoparticles (LNPs). *In vitro* expression, innate immune activation, immunogenicity and protective efficacy were evaluated in cell systems and mouse models.

**Results:**

mRNA-BMEPu was efficiently expressed in vitro as soluble oligomers and triggered innate immune activation in human macrophages. In C57BL/6 mice, LNP-BMEPu elicited robust binding and neutralizing antibodies against the ancestral virus and antigenically distant Omicron subvariants. Vaccination also induced strong and polyfunctional CD8⁺ T cell and T follicular helper responses that persisted over time. In K18-hACE2 transgenic mice, immunization conferred complete protection against lethal SARS-CoV-2 challenge, with effective control of viral replication and reduced lung inflammation.

**Discussion:**

These results support CoV2-BMEPu as a next-generation multi-epitope mRNA vaccine candidate capable of inducing broad, durable and protective immunity against current and emerging SARS-CoV-2 variants.

## Introduction

COVID-19, caused by severe acute respiratory syndrome coronavirus 2 (SARS-CoV-2), has led to over 779 million confirmed cases and more than 7 million deaths globally as of February 2026 (https://covid19.who.int). First-generation vaccines, particularly those based on messenger RNA (mRNA) technology such as BNT162b2 and mRNA-1273, significantly reduced severe disease and mortality. However, they failed to induce sterilizing immunity and required repeated booster doses due to waning effectiveness and the continuous emergence of immune-evasive viral variants (1–3). Although a variety of vaccine platforms have been developed, the vast majority of authorized vaccines, including updated formulations, rely exclusively on the Spike (S) glycoprotein, a highly immunogenic but rapidly evolving antigen. Mutations within Spike, especially in the receptor-binding domain (RBD), can compromise vaccine-induced neutralizing antibodies and limit the breadth and durability of protection (4, 5). In contrast, serological profiling and epitope-mapping studies in SARS-CoV-2 convalescent individuals have identified conserved and immunodominant antigenic regions in other structural proteins, including Membrane (M) and Nucleocapsid (N), as well as conserved segments within the RBD that overlap with structurally defined broadly neutralizing antibody (bNAb) epitope clusters. These findings support the development of next-generation vaccine strategies that incorporate conserved regions from multiple viral proteins to elicit broader and more durable immunity against antigenically diverse variants of concern (VOCs) (6–13).

Previously, we designed and preclinically evaluated CoV2-BMEP, a synthetic multi-patch immunogen composed of conserved antigenic regions derived from SARS-CoV-2 structural proteins. This construct elicited robust immune responses and partial protection (33%) in susceptible K18-hACE2 transgenic mice when delivered via DNA or MVA vectors (14). However, RBD regions were intentionally excluded due to concerns about sequence variability. While this strategy maximized sequence conservation, it did not account for RBD segments that are now recognized as key targets of bNAbs and central to effective humoral immunity.

In the present study we report an updated immunogen, CoV2-BMEPu, designed through refined antigen selection informed by recent immunological data from SARS-CoV-2 convalescent and vaccinated individuals. BMEPu integrates immunoprevalent B and T cell epitopes from the S, M and N proteins together with selected conserved RBD regions associated with bNAbs and cross-reactive responses. To enhance antigen presentation and immunogenicity, the construct was engineered as a secreted trimeric protein via fusion with a T4 fibritin fold on domain to promote a multimeric architecture. This refined design aims to broaden the immune response while improving durability and coverage against emerging SARS-CoV-2 variants. Here we evaluate the immunogenicity and protective efficacy of CoV2-BMEPu delivered as an mRNA vaccine formulated in lipid nanoparticles (LNP-BMEPu), with the aim of advancing a broadly protective next-generation vaccine strategy with universal potential against SARS-CoV-2.

## Materials and methods

### Cells

HeLa (ATCC Cat# CCL-2) and Vero-E6 (ATCC Cat# CRL-1586) cells were grown in Dulbecco’s Modified Eagle’s Medium (DMEM; Sigma-Aldrich, St. Louis, MO, USA) supplemented with 2 mM L-glutamine, 100 U/mL penicillin/100 µg/mL streptomycin, 0.1 mM non-essential amino acids (all from Sigma-Aldrich), 0.5 μg/mL amphotericin B (Fungizone; Gibco-Thermo Fisher, Waltham, MA, USA) and 10% heat-inactivated fetal bovine serum (FBS; Sigma-Aldrich). The human THP-1 monocyte cell line (ATCC Cat# TIB-202) was cultured in suspension in Roswell Park Memorial Institute-1640 medium (RPMI-1640; Sigma-Aldrich) supplemented as above, and differentiated into macrophages by incubation with 120 ng/mL phorbol 12-myristate 13-acetate (PMA; Sigma-Aldrich) for 48 h. All cell lines were maintained at 37 °C in a humidified atmosphere containing 5% CO_2_.

### Viruses

The SARS-CoV-2 strain MAD6 (kindly provided by Prof. Luis Enjuanes and Dr. José M. Honrubia, CNB-CSIC, Madrid, Spain) is a virus isolated from a nasopharyngeal swab from a 69-year-old male COVID-19 patient from Hospital 12 de Octubre (Madrid, Spain) as previously described (15). The SARS-CoV-2 Omicron VOCs XBB1.5, XBB1.16, KP.3 and JN.1 were kindly provided by Dr. Juan García-Arriaza (CNB-CSIC, Madrid, Spain). The different SARS-CoV-2 viral stocks were propagated in permissive VeroE6 TMPR cell line, under biosafety level-3 (BSL-3) conditions, following established protocols, and viral titers were determined by plaque assay.

### *In silico* design and immunoinformatic characterization of CoV2-BMEPu

The updated BMEPu immunogen was designed using an evidence-driven, multi-criteria strategy primarily guided by immunological data derived from SARS-CoV-2 convalescent individuals and supported by observations in vaccinated cohorts (6, 13, 16–27). Antigenic patches were selected based on published human serological and T cell epitope-mapping studies identifying regions that elicited robust IgG and IgA responses, as well as CD4^+^ and CD8^+^ T cell responses associated with durable immune memory (**Table 1**). Particular emphasis was placed on conserved regions within the Spike RBD corresponding to antigenic clusters targeted by bNAbs, as defined by structural and antibody escape-mapping studies (16–18, 28). The selected RBD-derived patches (S2-S6; aa 332-526) overlap conserved Class 3 and Class 4 antigenic sites located on the RBD core, as well as structurally constrained regions within the receptor-binding motif (RBM) associated with Class 1/2 antibody binding. Although these regions do not represent minimal conformational bNAb epitopes, several linear segments within these domains have been experimentally associated with antibody-mediated neutralization or mapped within neutralization-sensitive antigenic sites. In addition, the selected RBD patches encompass domains reported to elicit both humoral and cellular immune responses in convalescent cohorts (13, 16–19, 22, 24).

**Table 1:** Evidence-based selection of multi-epitope patches included in CoV2-BMEPu.

Patch ID^a^	Viral protein	Amino acid position^b^	Immune association (eeported in humans)	Representative supporting evidence
S1	Spike (NTD)	45–60	Linear B cell epitope; IgG/IgA reactivity in convalescent individuals	(19, 20, 24, 26)
S2	Spike (RBD core)	332–362	Conserved RBD core; Class 3/4 bNAb regional cluster; CD4^+^/CD8^+^ T cell reactivity	(13, 16–18, 22)
S3	Spike (RBD core)	393–414	RBD core epitope cluster; T cell responses in convalescent individuals	(17, 18, 22)
S4	Spike (RBD core–RBM boundary)	420–437	Core-adjacent region with bNAb regional overlap; CD4^+^ responses	(16)
S5	Spike (RBM)	454–470	RBM region targeted by Class 1/2 antibodies; immunodominant B cell responses	(16, 18, 19, 24)
S6	Spike (post-RBM core-proximal)	508–526	Conserved structural region; CD4^+^/CD8^+^ T cell responses	(17, 22)
S7	Spike (S1)	551–565	Reported CD4^+^ T cell epitope; antibody reactivity	(22, 24)
S8	Spike (S2)	816–835	Conserved CD8^+^ T cell epitope; cross-variant reactivity	(21, 25)
S9	Spike (S2)	893–915	Conserved S2 T cell responses; memory-associated	(21)
S10	Spike (S2)	1146–1165	S2 region implicated in T cell responses and conserved immunity	(20, 21)
M1	Membrane	1–20	Immunodominant CD4^+^/CD8^+^ T cell epitope in convalescent individuals	(13, 22)
M2	Membrane	201–220	Conserved T cell epitope; cross-reactive memory	(21, 22)
N1	Nucleocapsid	151–176	Strong CD4^+^/CD8^+^ T cell responses; long-term memory	(22)
N2	Nucleocapsid	217–231	T cell reactivity; immunodominant region	(22, 24)
N3	Nucleocapsid	386–408	Conserved CD8^+^ epitope; durable T cell immunity	(13, 22)

^a^ This nomenclature refers to peptide patches and not to viral protein subunits; ^b^Positions refer to amino acid numbering based on the SARS-CoV-2 Wuhan-Hu-1 reference sequence (NC_045512). Selected RBD-derived patches (S2–S6; in gray) overlap regionally with structurally defined broadly neutralizing antibody clusters (Classes 1–4) identified in structural and escape-mapping studies. RBM, receptor-binding motif.

Regions derived from the S, M and N proteins were included based on consistent reports of conserved CD4^+^ and CD8^+^ T cell responses across variants of concern (13, 20–22, 24, 25) and evidence of durable memory responses in convalescent individuals (13, 21, 22, 25).

**Figure 1:**
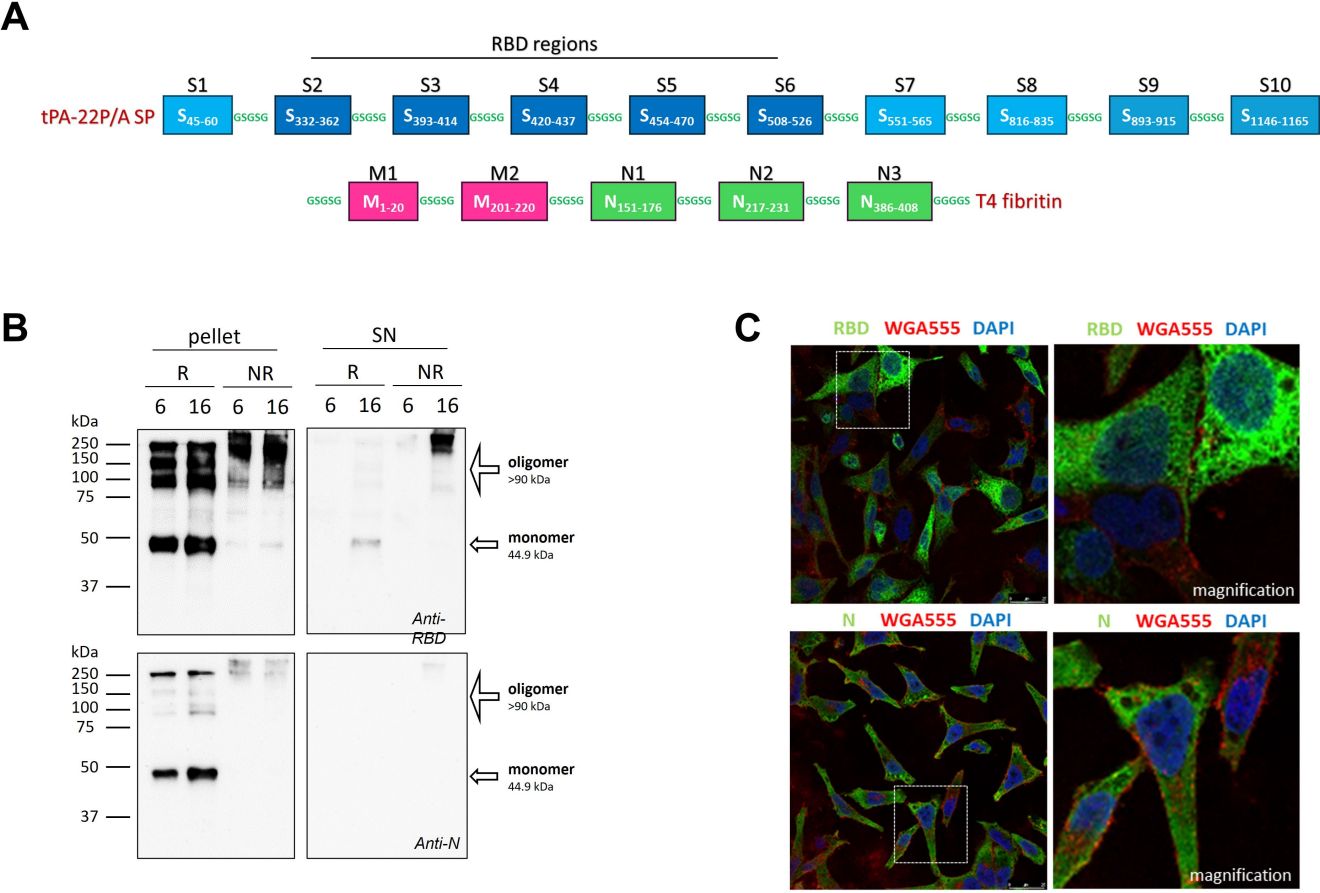
*In vitro* characterization of the mRNA encoding the multi-epitope CoV2-BMEPu protein. **(A)** Schematic representation of the chimeric multi-epitope CoV2-BMEPu protein, including epitope patches derived from the S, M and N proteins, the tissue plasminogen activator signal peptide (tPA-22P/A SP) and the T4 fibritin foldon domain. **(B)** Western-blot analysis of CoV2-BMEPu protein expression in HeLa cells transfected with 2 µg of mRNA-BMEPu at 6 and 16 h post-transfection (h.p.t.). Cellular pellets (pellet) and supernatants (SN) were analyzed under reducing (R) and non-reducing (NR) conditions using anti-RBD (upper panels) or anti-N (lower panels) antibodies. **(C)** Confocal microscopy analysis of HeLa cells at 6 h.p.t. Cells were labeled with WGA555 and stained with anti-RBD or anti-N antibodies (Alexa Fluor 488). Nuclei were stained with DAPI. Representative images are shown; insets correspond to magnified regions.

The final construct includes 15 epitope patches: 10 from the S protein (7 from S1 and 3 from S2), 2 from M and 3 from N (**Table 1**). Patches were joined using GSGSG linkers (**Figure 1A**). A tissue plasminogen activator signal peptide (tPA-22P/A SP) was added at the N-terminus to direct the protein into the classical endoplasmic reticulum–Golgi secretory pathway, and a T4 fibritin foldon domain was fused at the C-terminus to promote protein trimerization (27).

Predicted HLA class I and class II binding capacity of the selected BMEPu patches was evaluated using the Immune Epitope Database (IEDB) analysis resource. Class I binding predictions were performed with NetMHCpan (IEDB default implementation) across the IEDB ‘most frequent HLA-A and HLA-B’ reference allele panel, classifying. peptides with percentile rank ≤0.5% as strong binders and ≤2% as weak binders. Class II binding predictions were generated using the IEDB-recommended MHC-II binding predictor across the ‘most frequent 26 HLA-DR alleles’ reference panel, applying thresholds of ≤2% (strong binders) and ≤10% (weak binders). Strong binders therefore constitute a subset of the corresponding relaxed-threshold binders. Projected population coverage was calculated using the IEDB Population Coverage tool based on all predicted peptide–HLA combinations meeting the relaxed percentile rank thresholds (≤2% for class I; ≤10% for class II). These analyses were used to validate the global immunogenetic breadth of the selected antigenic regions rather than to guide their initial selection. Detailed binding profiles for each patch, including the number of predicted binders per HLA allele, best percentile rank, IC50 values (class I) and representative core sequences (class II), are provided in **Supplementary Table 1** and **Supplementary Table 2**, while population coverage estimates are reported in **Supplementary Tables 3** and **Supplementary Table 4**.

Physicochemical properties of the final construct were evaluated using the ProtParam tool and ExPASy Aldente Peptide Mass Fingerprinting tool, while antigenicity and allergenicity were assessed using ANTIGENpro and AllerHunter, respectively.

### *In vitro* transcription of mRNA-BMEPu

The synthetic mRNA encoding CoV2-BMEPu (mRNA-BMEPu) was produced by *in vitro* transcription at Vrije Universiteit Brussel (Belgium) using the proprietary plasmid backbone pLMCT (29). The coding sequence of CoV2−BMEPu was flanked by optimized 5′ and 3′ untranslated regions (UTRs) derived from human β-globin, which enhance mRNA stability and translation efficiency in mammalian cells (30, 31). The plasmid template also contained a poly(A) tail of 100 nucleotides. Full-length mRNA-BMEPu was synthesized from the linearized pLMCT-BMEPu plasmid using a T7 RNA polymerase-based transcription system incorporating N1−methyl−pseudouridine instead of uridine to reduce innate immune activation and improve translational efficiency (32). Following transcription, mRNA was purified by high−performance liquid chromatography to remove double-stranded RNA and transcriptional impurities. RNA integrity and size were verified by denaturing formaldehyde agarose gel electrophoresis and UV spectrophotometric analysis prior to LNP formulation. Protein expression and cellular localization were assessed by Western-blot and confocal microscopy.

### Formulation and physicochemical characterization of LNP-BMEPu

LNPs were formulated using a microfluidic solvent-displacement method. Lipids ALC-0315 (ionizable lipid), DSPC, cholesterol and ALC-0159 (PEG-lipid) were dissolved in ethanol and mixed with mRNA in citrate buffer (10 mM, pH 4) using a NanoAssemblr microfluidic system. Mixing was performed at a flow rate ratio (FRR) of 3:1 (aqueous-to organic) and a total flow rate of 9 mL/min, following previously described principles for LNP-mRNA vaccine formulation (33). Lipids were combined at a molar ratio of 48.4:9.8:40:1.8 (ALC-0315:DSPC:cholesterol:ALC-0159), consistent with clinically validated LNP compositions for mRNA delivery (34). Following assembly, LNP-BMEPu was diafiltered into PBS, concentrated and sterile−filtered through 0.22-µm membranes.

Particle size, polydispersity index (PDI) and zeta potential were measured using a Zetasizer Nano ZS (Malvern Instruments, UK). mRNA concentration and encapsulation efficiency were determined using the Quant-iT RiboGreen RNA assay (Invitrogen, Carlsbad, CA, USA) and confirmed by denaturing formaldehyde agarose gel electrophoresis (**Supplementary Figure 1A**). **Supplementary Figure 1B** shows BMEPu subcellular localization in transfected HeLa cells.

### *In vitro* expression analysis

#### Western-blot and confocal microscopy analyses

HeLa cells were seeded at a density of 1.5 × 10^5^ cells per well in 24-well plates, corresponding to approximately 70–80% confluence at the time of transfection, and transfected with 2 µg of naked mRNA-BMEPu using Lipofectamine 2000 (Invitrogen) or 5 µg of LNP-BMEPu. At 6- or 16-hours post-transfection (h.p.t.), cell lysates and supernatants were collected for Western-blot using a rabbit anti-RBD antibody (1:1000; GeneTex Cat# GTX135385, Irvine, CA, USA) or a rabbit anti-N antibody (Sino Biological Cat# 40588-RA84, Beijing, China). For SDS-PAGE analysis, samples were mixed with Laemmli buffer (Tris-HCl 50 mM pH 6.8 - 10% glycerol - 0.1% bromophenol blue - 2% SDS) containing β-mercaptoethanol, and electrophoresis and nitrocellulose membrane transfer were performed under standard conditions. Native-PAGE was carried out using non-denaturing conditions and avoiding prior heat denaturation of the samples to preserve oligomeric assemblies. The presence of the C-terminal T4 fibritin foldon domain confers high oligomeric stability, which can result in partial retention of oligomeric species under reducing SDS-PAGE conditions.

For confocal microscopy, transfected cells were stained with Alexa Fluor 555-conjugated wheat germ agglutinin (WGA 1:500; Thermo Fisher), fixed, permeabilized and incubated with anti-RBD (1:200) or anti-N (1:200) antibodies. Detection was performed using Alexa Fluor 488-conjugated goat anti-rabbit IgG (1:500; Molecular Probes Cat# A-11094, Eugene, OR, USA). Leica STELLARIS 5 Cryo Confocal Light Microscope and the specialized software LasX (Leica Microsystems, Wetzlar, Germany) were used for the acquisition of cellular optical sections and for image recording and processing, respectively.

#### Size exclusion chromatography

To assess the oligomeric state of the BMEPu protein, HeLa cells were seeded in three 150-mm tissue culture dishes and transfected with 25 µg of naked mRNA-BMEPu using Lipofectamine 2000 (Invitrogen). After 16 hours, culture supernatants were clarified by centrifugation (3,000 rpm, 20 min), filtered and concentrated to ~0.5 mL. Samples were loaded onto a Superdex 200 10/300 GL column (Cytiva AKTA, Barcelona, Spain) pre-equilibrated with PBS. Fractions (0.5 mL) were collected and analyzed by native Western-blot using Criterion TGX gels (Bio-Rad, Hercules, CA, USA). Band intensities corresponding to different oligomeric forms were quantified with Image Lab 6.1 software (Bio-Rad) to generate elution profiles. Estimated molecular weights of monomeric and oligomeric forms were calculated based on the primary amino acid sequence and structural prediction, as no prior literature data are available for this multi-patch epitope construct, and were estimated by comparison with native protein standards (Sigma-Aldrich), identifying monomer (~45 kDa), trimer (~135 kDa) and higher-order oligomers (>270 kDa).

#### Nano differential scanning fluorimetry analyses

Thermal stability of the BMEPu protein was analyzed using a Prometheus NT.48 system (NanoTemper Technologies GmbH, Munich Germany). Samples were subjected to a linear temperature gradient, and intrinsic fluorescence changes were recorded to determine the inflection temperature (Ti), indicative of protein unfolding. Thermal unfolding was independently assessed by nanoDSF, which monitors temperature-induced conformational changes in solution and is not directly comparable to detergent-based electrophoretic conditions.

### Peptides

The SARS-CoV-2 overlapping peptide pools (OLP) used for *in vivo* studies include S1 (158 peptides), S2 (157 peptides), M (53 peptides), N (102 peptides) and RBD (53 peptides) (JPT Peptide Technologies GmbH, Berlin, Germany). They covered the full-length sequences of the SARS-CoV-2 S, M, N and RBD proteins incorporated into the CoV2-BMEPu construct as consecutive 15-mer peptides, overlapping by 11 amino acids.

### Mouse immunizations

To evaluate immunogenicity, two groups of female C57BL/6JOlaHsd mice (n = 10 per group) purchased from Envigo Laboratories (Sant Feliu de Codines, Barcelona, Spain) were immunized intramuscularly (i.m.) on day 0 and 28 with 10 μg of LNP-BMEPu (G1) or LNP-Luc as control (G2) to exclude effects from the mRNA platform. The 10 µg dose was selected based on established dosing ranges commonly used in murine preclinical studies of LNP-formulated mRNA vaccines (33–35). Five mice per group were sacrificed on day 38 and 80 and spleens were collected to assess T cell responses by intracellular cytokine staining (ICS) assay. Blood samples were collected on day 21, 38 and 80 for serological analysis. Animals were housed at Centro Nacional de Biotecnología (CNB), Consejo Superior de Investigaciones Científicas (CSIC) (Madrid, Spain).

For efficacy studies, female K18-hACE2 transgenic mice (n = 5 per group) purchased from Jackson Laboratory (Bar Harbor, ME, USA) were immunized i.m. on day 0 and 28 with 10 μg of LNP-BMEPu (G1) or PBS as control (G2) to define an unvaccinated baseline, enabling comparison of disease severity and mortality. Blood was collected on day 21 and 49. On day 62 mice were challenged intranasally (i.n.) with 10^5^ PFU of SARS-CoV-2 MAD6 under isoflurane anesthesia. Animals were monitored daily for weight loss, clinical signs and survival for 12 days. Mice exceeding 25% weight loss were euthanized per humane endpoint criteria. Lung tissues were collected at the time of euthanasia: day 7 post-infection for PBS controls (humane endpoint) and day 12 post-infection for vaccinated mice (study completion/survival monitoring). All procedures were conducted in BSL-3 facilities at Centro de Investigación en Sanidad Animal (CISA), Instituto Nacional de Investigación y Tecnología Agraria y Alimentaria (INIA-CSIC) (Valdeolmos, Madrid, Spain).

### Ethics statement

Experimental protocols involving C57BL/6JOlaHsd and K18-hACE2 transgenic mice were reviewed and approved by the Ethical Committee for Animal Experimentation (CEEA) at CNB-CSIC and CISA-INIA and by the Animal Protection Authority of the Comunidad de Madrid, under permit numbers PROEX 169.4/20 and 161.5/20. All animal procedures were conducted in accordance with EU guidelines and Spanish legislation (Royal Decree RD 53/2013).

### Binding and neutralizing antibody assays

SARS-CoV-2-specific binding antibodies (BAbs) were measured by Enzyme-Linked Immunosorbent Assay (ELISA) as previously described (15). Briefly, individual sera from immunized mice were 3-fold serially diluted in duplicates and incubated with 2 μg/mL of recombinant S or RBD proteins. Levels of SARS-CoV-2 S- and RBD-specific total IgG BAbs were indicated as the last serum dilution yielding an optical density at 450 nm (OD450) three times above the mean of the control group (end-point titer).

Neutralizing antibody titers were determined by microneutralization test (MNT) using live SARS-CoV-2 (MAD6 and VOCs) in BSL-3 facilities at CNB-CSIC, as previously described (36). Titers were expressed as IC50 values calculated by nonlinear regression (log[agonist] vs. normalized response) using GraphPad Prism v10.1.0 Software (San Diego, CA, USA), with 95% confidence intervals.

### Multiparametric ICS analysis of SARS-CoV-2-specific effector and Tfh responses

To evaluate the magnitude, phenotype and polyfunctionality of SARS-CoV-2-specific T cell responses, 3 × 10^6^ erythrocyte-depleted splenocytes per well were seeded in 96-well plates in complete RPMI-1640 medium supplemented with 2 mM L-glutamine, 100 U/mL penicillin/100 μg/mL streptomycin, 10 mM HEPES, 0.01 mM β-mercaptoethanol and 10% FBS. For conventional T cell analysis, cells were stimulated *ex vivo* for 6 hours with 1 μg/mL of S1, S2, M or N peptide pools, or 5 μg/mL of RBD peptide pool, and anti-CD107a-FITC (BD Biosciences Cat# 553793, San Jose, CA, USA) in the presence of monensin (1X; Invitrogen) and GolgiPlug (1 μL/mL; BD Biosciences) added since the beginning of the stimulation period. To assess T follicular helper (Tfh) responses, splenocytes were stimulated *ex vivo* for 6 hours with 1 μg/mL of (S1+S2) peptide pools plus 5 μg/mL of recombinant S protein, or 2 μg/mL of RBD peptide pool plus 5 μg/mL of recombinant RBD protein, and anti-CD154 (CD40L)-PE (BD Biosciences Cat# 561719) in the presence of monensin (1X; Invitrogen) and GolgiPlug (1 μL/mL; BD Biosciences) added two hours after the start of the stimulation period. For both analyses, RPMI alone was used as the unstimulated control.

After stimulation, cells were stained with surface markers, fixed, permeabilized (Cytofix/Cytoperm kit, BD Biosciences) and stained intracellularly. Dead cells were excluded using LIVE/DEAD Fixable Violet Dead Cell Stain kit (Invitrogen). For conventional T cells, antibodies included CD3-PE-CF594 (BD Biosciences Cat# 740268), CD4-APC-Cy7 (BD Biosciences Cat# 553050), CD8-V500 (BD Biosciences Cat# 612898), IL-2-APC (BioLegend Cat# 503837), IFN-γ-PE-Cy7 (BD Biosciences Cat# 557649) and TNF-α-PE (Thermo Fisher Scientific Cat# 12-7321-82). For Tfh cells, antibodies included CD4-Alexa700 (BD Biosciences Cat# 563232), CD8-V500, CXCR5-PE-CF594 (BioLegend Cat# 145534), PD-1-APC-eFluor780 (Invitrogen Cat# 47-9985-82), CD19-SPRD (Southern Biotech Cat# 1575-13), IL-21-APC (Thermo Fisher Scientific Cat# 17-7211-80), IFN-γ-PE-Cy7 and IL-4-Alexa488 (BD Biosciences Cat# 557990).

Samples were acquired on a GALLIOS flow cytometer (Beckman Coulter, Brea, CA, USA) and analyzed using FlowJo software (Version 10.4.2; Tree Star, Ashland, OR, USA). Lymphocyte-gated events ranged from 10^5^ to 5 × 10^5^. Boolean gating was applied to identify all cytokine combinations. Background responses from RPMI controls were subtracted from stimulated samples. The gating strategies used to identify SARS-CoV-2-specific CD4^+^, CD8^+^ and Tfh-associated CD4^+^ T cell responses are shown in **Supplementary Figure 4**.

### Analysis of SARS-CoV-2 viral yields by plaque assay

Lung samples from K18-hACE2 mice were processed and evaluated for SARS-CoV-2 infectious virus using a plaque assay previously reported (37). Briefly, serial 10-fold dilutions of homogenized lung tissue were added in triplicate to Vero-E6 cell monolayers seeded in 12-well plates. After 1 h of adsorption, the inoculum was removed and the plates were overlayed with semisolid medium (DMEM 2X-4% FBS: Avicel^®^ RC-591 -DuPont Nutrition Biosciences ApS, Kongens Lyngby, Denmark) and incubated at 37 °C in 5% CO_2_. After 4 days, cells were fixed with 10% formaldehyde (Sigma-Aldrich) for 1h, the supernatant was removed and plaques were observed by the addition of 0.5% crystal violet (Sigma-Aldrich) and counted to calculate plaque-forming units per gram of lung tissue (PFU/g).

### Analysis of SARS-CoV-2 RNA and cytokines by quantitative RT-PCR

Total RNA was extracted using the RNeasy^®^ Mini Kit (Qiagen, Hilden, Germany) from THP-1-derived macrophages or homogenized lung tissue (Miltenyi gentleMACS Dissociator; Miltenyi Biotec, Bergisch Gladbach, Germany) in RLT buffer with β-mercaptoethanol. RT-qPCR was performed using TaqMan or SYBR Green chemistry with the NZYSpeedy RT-qPCR Green Kit (NZYTech, Lisbon, Portugal) or Verso 1-step RT-qPCR Kit (Thermo Fisher) on a QuantStudio™ 5 system (Life Technologies; Waltham, MA, USA). Gene expression was normalized to 28S rRNA and calculated using the 2^-^ΔΔCt method. Results were expressed as log_2_ fold change relative to mock (fold changes are calculated relative to mock-transfected cells for THP-1 cells or relative to PBS-injected control mice in lung tissue after *in vivo* challenge. No empty LNPs were included in these experiments). In THP-1 cells, expression of cytokine and innate immune genes (e.g., *TNFα, IL6, IL8, IL12, CCL2, CXCL10, TRIM5α* and *IFIT1*, among others) was analyzed. In lung samples, SARS-CoV-2 genomic (*RdRp*) and subgenomic (*E*) RNA levels were quantified. Probes and primer sequences used are listed in **Supplementary Table 5**.

### Data analysis and statistics

RT-qPCR data were analyzed using repeated measures two-way ANOVA with Dunnett’s post-test. Binding antibody titers were compared using ordinary two-way ANOVA. Neutralizing antibody titers and viral yields were analyzed by two-way ANOVA on log-transformed data with Tukey’s post-test. For the statistical analysis of ICS data, an approach that adjusts the values for the unstimulated controls (RPMI) and calculates the *p* values and confidence intervals was used (38). Only specific responses significantly higher than the corresponding RPMI values were represented. All values are background-subtracted. Weight loss was normalized to baseline (day 0) and analyzed by two-way ANOVA with interaction terms. Graphs and statistics were generated using GraphPad Prism v10.1.0 Software. Statistical significance was defined as: *, *p* < 0.05; **, *p* < 0.005; ***, *p* < 0.001; ****, *p* < 0.0001.

## Results

### Biophysical characterization and *in vitro* expression of mRNA-BMEPu

#### *In silico* characterization of the BMEPu immunogen

To assess the predicted biochemical and immunological properties of the rationally designed BMEPu antigen, we performed an *in silico* characterization. The construct (429 amino acids; ~45 kDa) exhibited favorable physicochemical properties, including a predicted isoelectric point (pI) of 5.89, an instability index of 27.23 (consistent with protein stability), an aliphatic index of 76.62 (suggesting thermostability), and a GRAVY score of −0.145, indicative of a predominantly hydrophilic profile. ANTIGENpro predicted the construct to be antigenic (score: 0.815), while AllergenFP classified it as non-allergenic.

Computational HLA binding analysis of the selected BMEPu patches indicated broad engagement of HLA class I and class II alleles, with several regions containing multiple predicted high-affinity binders (**Supplementary Tables 1**, **2**). Population coverage estimates indicated high global representation, reaching approximately 89% for HLA class I and 64% for HLA class II across major geographic regions (**Supplementary Tables 3**, **4**).

#### *In vitro* expression and oligomerization of mRNA-BMEPu

Expression of mRNA-BMEPu was assessed in HeLa cells after transfection with 2 µg of naked mRNA using Lipofectamine 2000 (Invitrogen). Western-blot analysis using an anti-RBD antibody confirmed efficient expression and secretion of a protein of the expected molecular weight (~45 kDa) in cell lysates (6 h.p.t.) and culture supernatants (16 h.p.t.), respectively (**Figure 1B**, upper panels). Despite the reducing conditions, we detected higher molecular weight species in cell lysates consistent with foldon-mediated oligomerization of the expressed protein. Under non-reducing conditions, additional higher molecular weight oligomers were observed at both time-points in cell lysates (**Figure 1B**, upper panels). Additional immunoblotting using an anti-N antibody confirmed the presence of N-derived regions within the expressed protein (**Figure 1B**, lower panels). Confocal microscopy revealed a diffuse cytoplasmic distribution of BMEPu with punctate accumulations, indicative of intracellular oligomerization (**Figure 1C**).

#### Oligomerization analysis of CoV2-BMEPu protein

**Figure 2:**
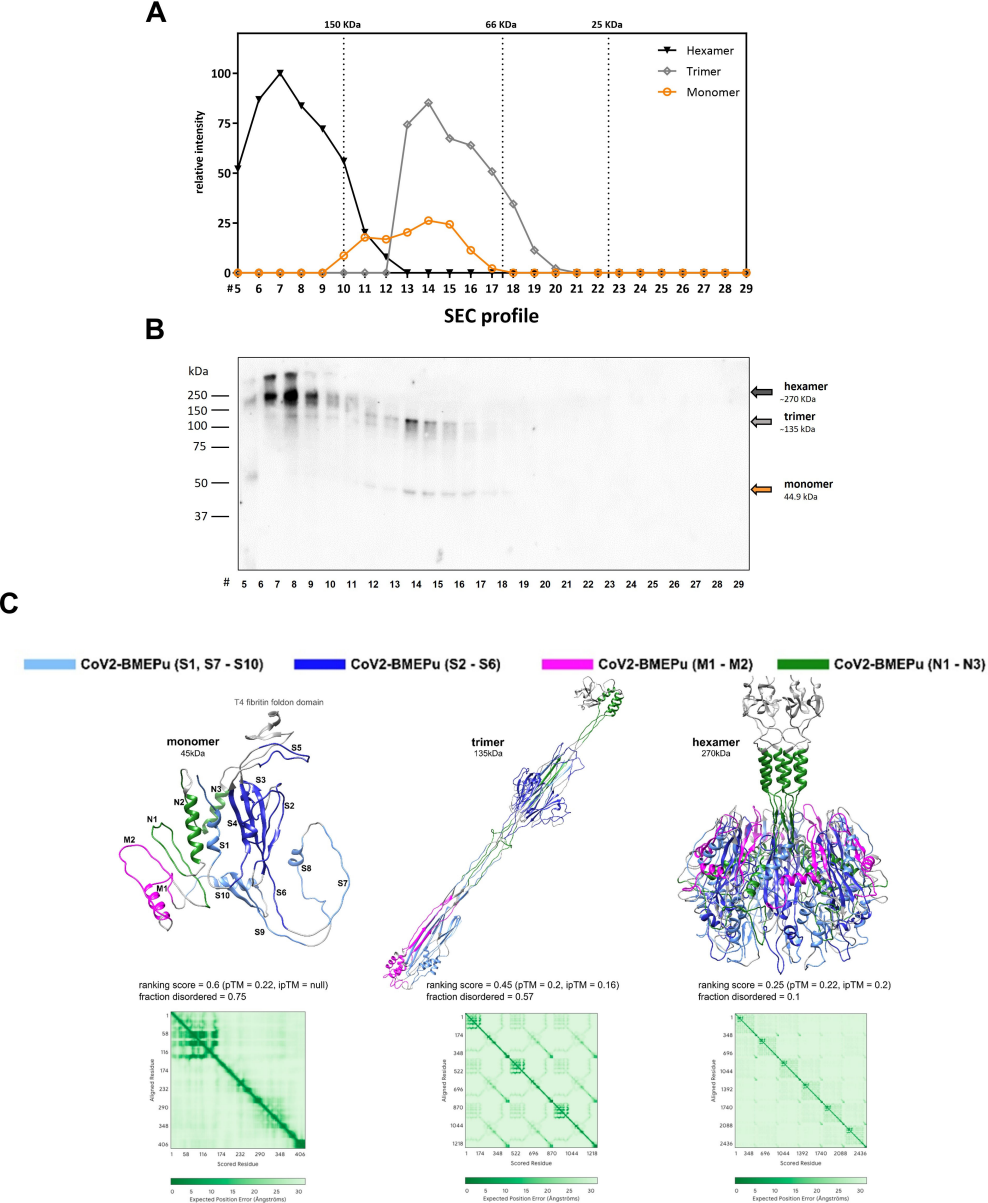
Oligomerization and structural analysis of the CoV2-BMEPu protein. **(A)** Size exclusion chromatography (SEC) profile of supernatants from HeLa cells transfected with 2 µg of mRNA-BMEPu. Quantification of protein abundance across SEC fractions indicates the presence of monomeric, trimeric and higher-order oligomeric forms. Molecular weight standards are indicated. **(B)** Collected fractions were analyzed by Native-PAGE and Western-blot using an anti-RBD antibody. **(C)** Three-dimensional structural models of CoV2-BMEPu predicted by AlphaFold 3. Epitope patches are annotated according to **Table 1**. Predicted aligned error (PAE) plots are shown for each oligomeric state. Structures were visualized using UCSF Chimera.

Size exclusion chromatography (SEC) of supernatants from mRNA-BMEPu-transfected HeLa cells revealed species consistent with monomeric (~45 kDa), trimeric (~135 kDa) and higher-order oligomeric forms (>270 kDa) (**Figure 2A**). Native PAGE and Western-blot using an anti-RBD antibody confirmed the presence of these forms (**Figure 2B**), while Coomassie staining validated protein identity and purity (**Supplementary Figure 2A**). NanoDSF analysis revealed distinct thermal transitions, consistent with the presence of thermally stable oligomeric species (**Supplementary Figure 2B**).

Structural models generated with AlphaFold 3 (39) (ModelArchive: ma-4tb8w, ma-ut8lv, ma-zt6is) revealed a β-sheet-rich N-terminal domain (S peptides) and an α-helix-rich disordered C-terminal domain (M and N peptides). Although predicted pTM (<0.5) and ipTM (<0.75) scores were modest, PAE plots showed structured epitopes S2–S6, with S5 (RBD) most exposed. ElliPro analysis (40) identified epitopes with high protrusion index (EPI > 0.7), including S2–S4, S9–S10, M1–M2, N3 (trimer) and S5, S7–S8, N3 (hexamer) (**Figure 2C**), supporting efficient epitope display.

### Innate immune activation by LNP-BMEPu

**Figure 3:**
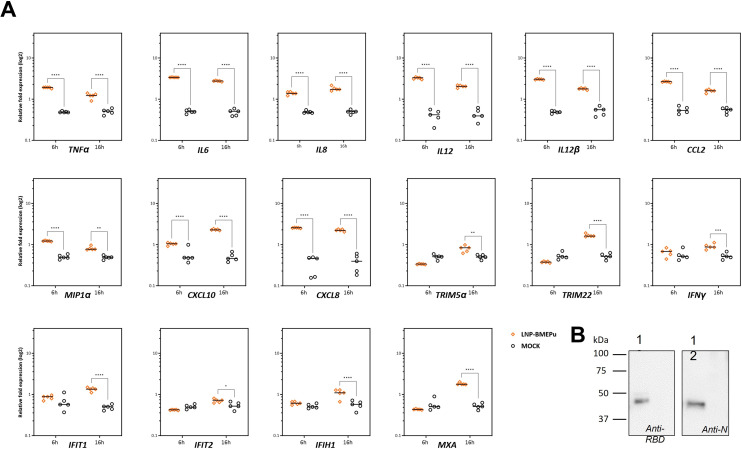
Innate immune activation induced by LNP-BMEPu in THP-1-derived macrophages. **(A)** Relative mRNA expression levels of pro-inflammatory cytokines and interferon-stimulated genes (ISGs) measured by RT-qPCR in THP-1-derived macrophages transfected with 5 µg of LNP-BMEPu at 6 and 16 h.p.t. Data are expressed as log_2_ fold change relative to mock-transfected cells. **(B)** Western-blot analysis confirming BMEPu protein expression in THP-1-derived macrophages after LNP-BMEPu transfection (1: LNP-BMEPu; 2: MOCK). MOCK: THP−1−derived macrophages incubated with the corresponding buffer without mRNA or LNP. Statistical analysis was performed using two-way ANOVA followed by Dunnett’s multiple comparisons test. *p < 0.05; **p < 0.005; ***p < 0.001; ****p < 0.0001.

To assess innate immune activation, THP-1-derived macrophages were mock-transfected or transfected with 5 µg of LNP-BMEPu and analyzed at 6 and 16 h.p.t. RT-qPCR analysis revealed a marked upregulation of key pro-inflammatory cytokines and interferon-stimulated genes (*ISGs*). Among these, *TNF-α, IL-6, IL-12, IL-12β* and *IFNγ* showed significant increases in transcript levels, indicating their central role in the innate response triggered by LNP-BMEPu (**Figure 3A**). Notably, *MXA*, a downstream effector of the RNA sensor IFIH1 (MDA5), together with canonical antiviral ISGs such as *IFIT1* and *IFIT2*, were significantly upregulated, consistent with activation of antiviral innate immune pathways. These findings indicate that LNP-BMEPu induces a potent innate immune response characterized by the induction of inflammatory cytokines and antiviral mediators. Western-blot analysis confirmed the expression of the BMEPu protein in THP-1 cells after LNP-BMEPu transfection (**Figure 3B**).

### SARS-CoV-2-specific humoral and cellular immune responses induced in C57BL/6 mice by homologous prime/boost immunization with LNP-BMEPu

**Figure 4:**
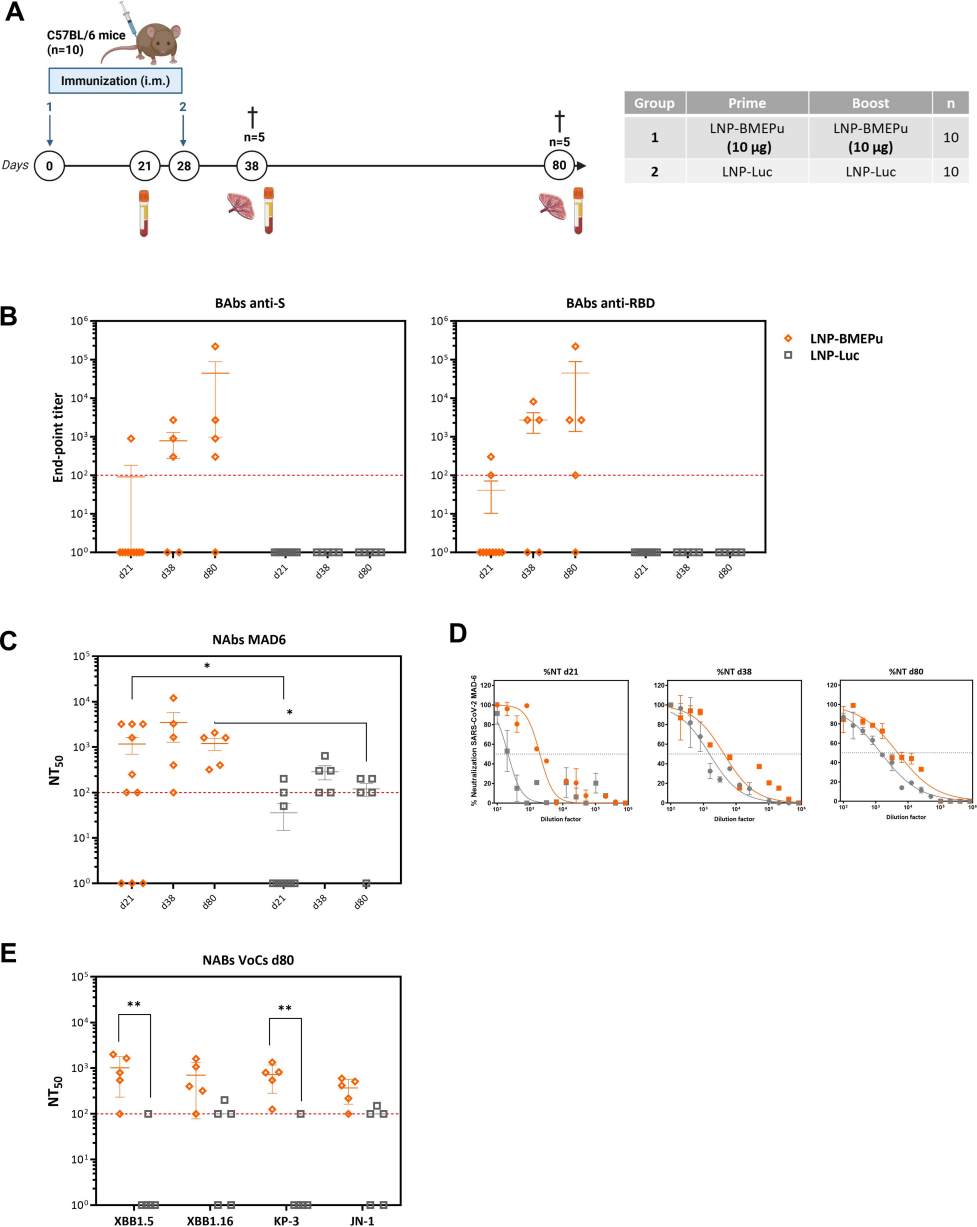
LNP-BMEPu homologous prime–boost vaccination induces robust humoral immune responses in C57BL/6 mice. **(A)** Immunization schedule. **(B)** SARS-CoV-2 S- (left panel) and RBD- (right panel)-specific IgG binding antibody titers measured by ELISA in individual serum samples collected at day 21, 38 and 80. Each symbol represents one animal; bars indicate geometric mean ± SD. The dashed red line indicates the lower limit of detection (LLD) of the ELISA assay. Samples with titers below LLD were plotted at the threshold value for visualization. **(C)** Neutralizing antibody titers against the ancestral MAD6 SARS-CoV-2 strain at the indicated time-points. **(D)** Representative neutralization curves obtained with sera against the MAD6 strain. **(E)** Neutralizing antibody titers against Omicron subvariants at day 80. Statistical analysis was performed using two-way ANOVA on log-transformed values followed by Tukey’s multiple comparisons test. *p < 0.05; **p < 0.005.

Following confirmation that LNP-BMEPu mediates expression of a soluble, oligomeric antigen and triggers innate immune activation *in vitro*, we next assessed its immunogenicity *in vivo*. For this, C57BL/6 mice received a homologous prime/boost regimen with LNP-BMEPu, while LNP-Luc was used as a formulation-matched negative control to account for potential innate/adjuvant effects of the LNP-mRNA platform and isolate BMEPu-specific adaptive responses. Humoral and cellular responses were evaluated at different time-points according to the immunization schedule depicted in **Figure 4A**.

#### LNP-BMEPu induces robust binding and neutralizing antibody responses

Sera collected at day 21 (post-prime), 38 (10 days post-boost) and 80 (52 days post-boost) were analyzed for SARS-CoV-2-specific IgG BAbs and NAbs. ELISA revealed significantly higher BAb titers against recombinant S and RBD proteins in LNP-BMEPu-immunized mice, whereas no detectable responses were observed in LNP-Luc control group (**Figure 4B**). Consistently, microneutralization assays demonstrated potent neutralizing activity against the ancestral MAD6 SARS-CoV-2 strain (**Figure 4C**), with clear dose-dependent neutralization profiles (**Figure 4D**). Neutralizing antibody responses were detectable following the prime-boost regimen and remained sustained at the late time-point (day 80). This timepoint was included to evaluate long-term persistence of the response rather than to capture the post-boost peak. In many vaccination settings, neutralizing antibody titers reach maximal levels shortly after boosting and subsequently contract and stabilize as the response transitions to a maintenance phase. Consistent with this kinetic pattern, neutralizing activity remained detectable at day 80 without further increase relative to day 38, supporting the persistence of functional antibody responses over time. In a small number of animals, neutralizing activity was detected despite low or undetectable BAb titers, likely reflecting methodological differences between assays. While ELISA measures antigen binding, microneutralization assays detect functional inhibition of viral infection and may reveal activity close to the ELISA detection threshold. At day 80, cross-neutralization of Omicron subvariants (XBB.1.5, XBB.1.16, KP.3 and JN.1) was detected, with NT_50_ titers up to 10³ (**Figure 4E**), indicating sustained and broad humoral immunity.

#### LNP-BMEPu induces robust and polyfunctional SARS-CoV-2-specific T cell responses in C57BL/6 mice

**Figure 5:**
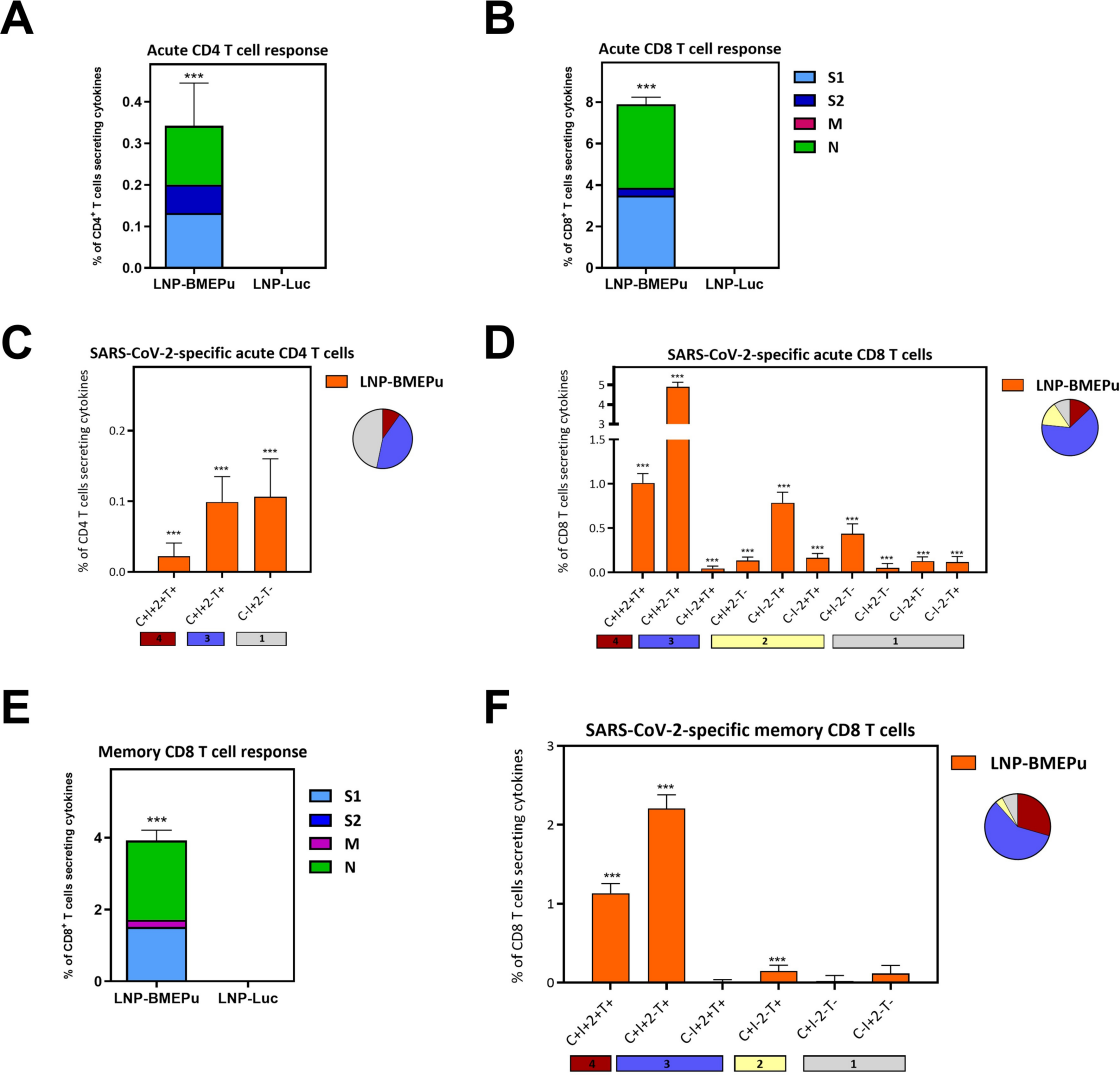
Magnitude, breadth and polyfunctional profile of the SARS-CoV-2-specific T cell responses induced by LNP-BMEPu. **(A–D)** SARS-CoV-2-specific T cell responses at day 38 (acute phase). **(A, B)** Magnitude of the overall SARS-CoV-2-specific CD4 **(A)** or CD8 **(B)** T cells in spleen from immunized mice at day 38. The overall response represents the sum of the percentages of the SARS-CoV-2-specific CD4 or CD8 T cells expressing CD107a and/or secreting IFN-γ and/or IL-2 and/or TNF-α. Data are background-subtracted. 95% CI is shown. **(C, D)** Polyfunctional profile of the overall SARS-CoV-2-specific CD4 **(C)** or CD8 **(D)** T cells. The positive combinations of the responses are indicated on the x axis, while the percentages of the functionally different cell populations within the total CD4 or CD8 T cells are represented on the y axis. Specific responses are grouped and color-coded based on the number of functions. C: CD107a; I: IFN-γ; 2: IL-2; T: TNF-α. **(E, F)** SARS-CoV-2-specific T cell responses at day 80 (long-term phase). Magnitude **(E)** and polyfunctional profile **(F)** of the overall SARS-CoV-2-specific CD8 T cells in spleen from immunized mice at day 80. ***p < 0.001.

To assess cellular immunity, splenocytes were harvested at day 38 (acute phase) and 80 (long-term phase) and stimulated *ex vivo* with SARS-CoV-2 peptide pools. At day 38, flow cytometry analysis revealed strong antigen-specific T cell responses, mainly mediated by CD8 T cells, particularly against S1 and N peptide pools (**Figures 5A, B**). No responses were detected in the LNP-Luc control group. CD4^+^ T cells showed a polyfunctional profile, with ~50% of responding cells expressing three or more functions (IFN-γ, IL-2, TNF-α, CD107a) (**Figure 5C**). CD8^+^ T cells exhibited an even higher degree of polyfunctionality, with >90% of cells co-expressing two to four effector markers. Dominant subsets included CD107a^+^IFN-γ^+^TNF-α^+^ and CD107a^+^IFN-γ^+^IL-2^+^TNF-α^+^ populations (**Figure 5D**). At day 80, CD4^+^ T cell responses declined below the detection threshold, whereas strong CD8^+^ T cell responses, particularly against S1 and N peptides, persisted (**Figure 5E**) with similar polyfunctionality (**Figure 5F**). RBD-specific CD4 and CD8 T cell responses were detected at both time-points, with a clear predominance of CD8^+^ T cells (**Supplementary Figure 3A**). Tfh-associated CD4^+^ T cell responses specific for S and RBD antigens were also detected during the acute phase, supporting the induction of germinal center associated helper responses that may contribute to the generation of long-lived humoral immunity (**Supplementary Figure 3B**).

### Protective efficacy of LNP-BMEPu vaccination in K18-hACE2 transgenic mice

**Figure 6:**
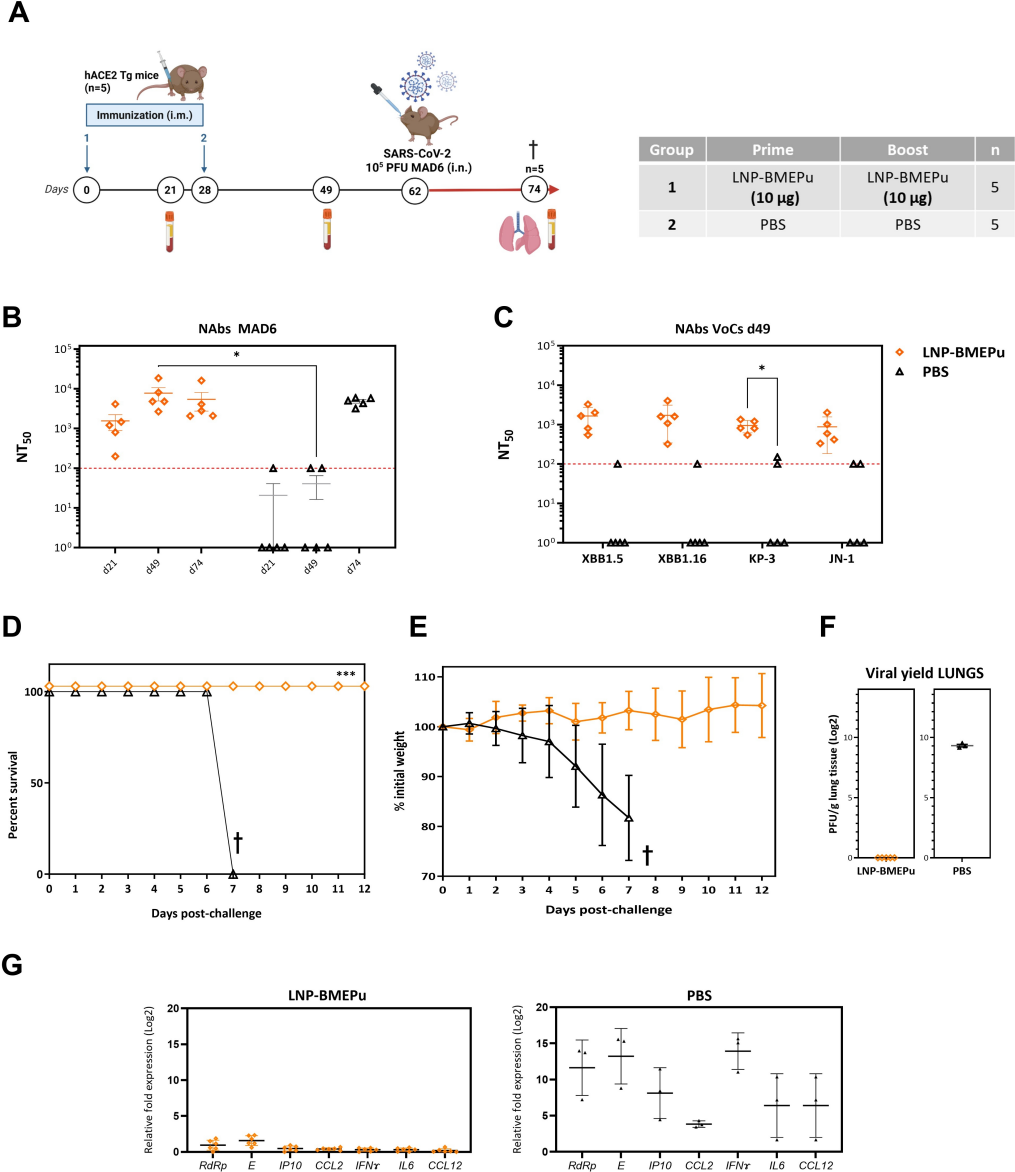
LNP-BMEPu vaccination confers full protection against lethal SARS-CoV-2 challenge in K18-hACE2 mice. **(A)** Immunization and challenge schedule. **(B)** Neutralizing antibody titers measured at day 21, 49 and 74. The red dotted line indicates the LLD. **(C)** Cross-neutralization of Omicron subvariants at day 49 post-boost. **(D)** Survival curves following SARS-CoV-2 challenge. **(E)** Body weight changes monitored for 12 days post-challenge. **(F)** Infectious viral titers detected in lung tissue by plaque assay at the time of euthanasia. **(G)** Relative expression of viral (*RdRp* and *E*) and inflammatory (IFNg, IL6, CCL2, CCL12 and IP10) transcripts in lung tissue measured by RT-qPCR. Lung samples were collected at the time of euthanasia (day 7 post-infection for PBS controls and day 12 for vaccinated mice). Statistical analysis was performed using two-way ANOVA on log-transformed values followed by Tukey’s or Dunnett’s multiple comparisons tests, as appropriate. †, sacrifice; *p < 0.05; ***p < 0.001.

To evaluate the protective efficacy of LNP-BMEPu, K18-hACE2 transgenic mice were immunized using a homologous prime/boost regimen and subsequently challenged with a lethal dose of live SARS-CoV-2 virus following the schedule indicated in **Figure 6A**. PBS-challenged animals served as the unvaccinated infection baseline for disease severity and survival comparisons.

#### SARS-CoV-2-specific humoral responses before and after viral challenge

Neutralizing antibody responses were evaluated in sera collected at day 21 post-prime (d21), day 21 post-boost (d49) and day 74 (end of study) using a microneutralization assay. As shown in **Figure 6B**, a single dose of LNP-BMEPu (d21) induced detectable neutralizing activity against the ancestral MAD6 SARS-CoV-2 strain, with NT_50_ titers exceeding 10³. These titers increased following the booster dose (d49), reaching approximately 10^4^, and remained detectable and stable after viral challenge (d74), indicating a sustained humoral response. In contrast, PBS-treated control mice only developed neutralizing antibodies at d74, 12 days after the virus challenge, likely reflecting antibody responses induced by active viral replication. Cross-neutralization assays at d49 showed that sera from LNP-BMEPu-immunized mice effectively neutralized the Omicron subvariants XBB.1.5, XBB.1.16, KP.3 and JN.1, with NT_50_ titers ranging from 10³ to 10^4^ (**Figure 6C**), supporting the cross-variant neutralizing potential of the vaccine-induced antibody response.

#### Protection against lethal challenge and viral control

To evaluate protective efficacy, the vaccinated K18-hACE2 transgenic mice were challenged with a lethal dose of SARS-CoV-2. Clinical outcomes were monitored over 12 days. As shown in **Figures 6D, E**, all control animals (G2) exhibited rapid weight loss and reached the humane endpoint by day 7 post-challenge (d7 p.c.), whereas 100% of LNP-BMEPu-vaccinated mice (G1) maintained stable body weight, showed no clinical signs and survived the entire observation period. To evaluate protective efficacy, K18-hACE2 transgenic mice were challenged with a lethal dose of SARS-CoV-2 and monitored for clinical outcomes over a 12-day period. As shown in **Figures 6D, E**, all control animals (G2) exhibited rapid weight loss and reached the humane endpoint by day 7 post-challenge (d7 p.c.), whereas 100% of LNP-BMEPu–vaccinated mice (G1) maintained stable body weight, showed no clinical signs and survived the entire observation period. Because PBS-treated control animals reached humane endpoints earlier in this lethal infection model, lung tissues were collected at the time of euthanasia (day 7 for PBS controls and day 12 for vaccinated animals). Consequently, viral measurements represent descriptive observations at the time of sampling rather than strictly time-matched comparisons between groups. Infectious virus was undetectable in the lungs of vaccinated mice at day 12, whereas high viral titers were detected in PBS control animals euthanized at their humane endpoint (day 7) (**Figure 6F**). Due to early mortality in the unvaccinated control group, only three PBS-treated animals were available for viral quantification. SARS-CoV-2 genomic *(RdRp*) and subgenomic (*E*) RNA levels measured by RT-qPCR in lung tissue showed no detectable viral transcripts in vaccinated mice at day 12, while PBS controls exhibited high viral RNA levels consistent with active replication (**Figure 6G**). Together, these findings are consistent with the survival and clinical outcomes observed and support the protective efficacy of LNP-BMEPu vaccination.

#### LNP-BMEPu vaccination mitigates SARS-CoV-2-induced pulmonary inflammation

Due to the well-established link between the upregulation of pro-inflammatory cytokines and COVID-19 severity, commonly referred to as a “cytokine storm” (41–44), we quantified the expression of inflammatory mediators in lung homogenates by RT-qPCR. We analyzed *IFNγ, IL-6, CCL2, CCL12* and *CXCL10/IP-10* as representative markers capturing key antiviral and inflammatory axes of the lung immune response. As shown in **Figure 6G**, pulmonary transcript levels of these mediators measured at euthanasia were low in LNP-BMEPu-vaccinated mice at day 12 post-challenge (left panel) whereas PBS-treated controls euthanized at day 7 exhibited markedly elevated expression levels (right panel). Consistent with the sampling framework described above, these measurements are presented descriptively within each group. Overall, the reduced inflammatory transcriptional profile observed in vaccinated animals is consistent with the absence of clinical disease and supports the conclusion that LNP-BMEPu vaccination mitigates pulmonary inflammatory responses typically associated with severe SARS-CoV-2 infection.

## Discussion

The continuous evolution of SARS-CoV-2, particularly the emergence of immune-evasive variants, has limited the effectiveness and durability of current Spike-based vaccines. While these strategies initially reduce disease severity, their reliance on a highly mutable antigen restricts their breadth of protection (23). As a result, there is a growing interest in next-generation vaccines that incorporate conserved epitopes and elicit coordinated humoral and cellular immune responses.

Multi-epitope immunogens represent a promising strategy to expand antigenic coverage. Several candidates, including UB-612, CoVac-1 and CoVepiT, have advanced to clinical evaluation, combining epitopes derived from structural and non-structural proteins to enhance T cell responses and cross-reactivity across variants (8–12). However, many of these platforms rely on peptide mixtures or require co-administration with adjuvants, increasing formulation complexity. In contrast, the CoV2-BMEPu immunogen described here provides a streamlined alternative: a single-component, mRNA-encoded trimeric protein that integrates conserved and functionally relevant regions from the S, M and N structural proteins of SARS-CoV-2 (13, 16, 19–26, 41).

The design of CoV2-BMEPu prioritizes naturally occurring immunogenic segments containing overlapping epitopes rather than the artificial concatenation of minimal peptides. This strategy facilitates physiological antigen processing and presentation, improves HLA coverage and preserves structural determinants relevant for B cell recognition. By maintaining the native immunological context of these regions, the construct captures overlapping B- and T-cell epitopes that may contribute to broader immune recognition while avoiding limitations associated with minimal epitope assembly. In addition, inclusion of the T4 fibritin foldon domain stabilizes the antigen in a multimeric conformation and supports multivalent epitope display, a feature that may promote B cell receptor cross-linking and efficient germinal center–associated responses (27, 45).

Our data demonstrate that LNP-BMEPu elicited a robust and balanced adaptive immune response, characterized by high titers of cross-neutralizing antibodies, polyfunctional CD8^+^ T cells and detectable Tfh responses. Functional CD4^+^ T cell help was inferred from the magnitude of the humoral responses and the detection of Tfh responses, which are indicative of germinal center–associated B cell responses (34, 35, 46). Notably, the inclusion of conserved RBD-derived regions likely contributed to the neutralizing activity observed against multiple Omicron subvariants. Similar observations have been reported for other peptide-based vaccine constructs that induce Th1-skewed cellular responses and neutralization breadth (11, 47–49). The persistence of functional T cell responses and cross-variant neutralization at day 80 further suggest sustained immune responses following vaccination.

Importantly, BMEPu vaccination conferred complete protection against lethal SARS-CoV-2 challenge in K18-hACE2 mice. Vaccinated animals maintained stable body weight, showed no detectable infectious virus in the lungs and exhibited markedly reduced pulmonary cytokine levels, which are surrogate indicators of reduced inflammation during severe infection (33, 41, 43, 44). Lung viral loads and cytokine measurements were obtained at the time of euthanasia (day 7 in controls vs day 12 in vaccinated mice), which prevents strictly time-matched quantitative comparisons between groups. Nevertheless, these observations are consistent with the survival outcomes and the absence of clinical disease in vaccinated animals and support both the protective efficacy and immunomodulatory properties of the vaccine platform.

Recent studies further support the rationale for conserved multi-epitope designs. Qin et al. demonstrated that a dual LNP-mRNA vaccine encoding the Omicron Spike together with more than 100 conserved T cell epitopes conferred complete protection in mice (50). Similarly, Invencao et al. reported that a DNA-based vaccine encoding 15 epitopes derived from S and N proteins elicited strong T cell responses (51). Unlike these approaches, CoV2-BMEPu preserves naturally occurring immunogenic regions containing overlapping B- and T-cell determinants while achieving antigenic complexity within a single molecular entity.

While further studies are needed to assess the durability of immune memory and validate efficacy in humans, our preclinical data position CoV2-BMEPu as a promising next-generation vaccine candidate. By integrating conserved immunogenic regions into a structurally stable trimeric antigen delivered through an mRNA platform, BMEPu illustrates how epitope-guided, single-molecule vaccine designs may help mitigate variant-driven immune escape. This strategy supports the development of broadly protective SARS-CoV-2 vaccines and may provide a conceptual framework for designing vaccines against other rapidly evolving viral pathogens.

## Data Availability

The raw data supporting the conclusions of this article will be made available by the authors without undue reservation.

## Supplementary material

Supplementary Figure 1 *In vitro* characterization of LNP-BMEPu particle. **(A)** mRNA concentration and estimated RNA encapsulation efficiency using RiboGreen (RG) and denaturing formaldehyde agarose gel assays (representative; left panel). Measurement of main physicochemical characteristics of LNP-BMEPu particles (right panel). **(B)** Subcellular distribution of BMEPu protein in HeLa cells transfected with 5 µg of LNP-BMEPu by confocal microscopy. Cells were fixed at 6 h.p.t. and labelled with WGA-555 and anti-RBD or anti-N antibodies (Alexa-488). The images on the right correspond to the magnification of the regions indicated by the discontinuous white square in the images on the left. Cells were stained with DAPI to detect cell nuclei.

Supplementary Figure 2 Oligomerization analysis of the CoV2-BMEPu protein. **(A)** Coomassie staining of Native-PAGE analysis of BMEPu under non-denaturing conditions, preserving higher-order oligomeric assemblies. **(B)** NanoDSF thermal unfolding profile of purified BMEPu protein, showing a cooperative unfolding transition at approximately 87 °C, indicative of high thermal stability in solution.

Supplementary Figure 3 **(A)** Magnitude and polyfunctional profile of the SARS-CoV-2 RBD-specific T cell response. Magnitude of the SARS-CoV-2 RBD-specific acute (left) or memory (right) CD4 or CD8 T cells in the spleens from immunized mice at day 38 and 80, respectively. The overall response represents the sum of the percentages of the SARS-CoV-2 RBD-specific CD4 or CD8 T cells expressing CD107a and/or secreting IFN-γ and/or IL-2 and/or TNF-α. Data are background-subtracted. 95% CI is shown. The polyfunctional profile of the SARS-CoV-2 RBD-specific T cells is represented below each figure. Specific responses are grouped and color-coded based on the number of functions. **(B)** Magnitude of the SARS-CoV-2-specific Tfh-associated CD4^+^ T cell responses in spleens from immunized mice at day 38 following stimulation with SARS-CoV-2 S or RBD peptide pools. The total response in each group represents the percentage of Tfh^+^ cells expressing IL-21 and/or IL-4 and/or IFN-γ and/or CD154 (CD40L). Data are background-subtracted. 95% CI is shown. ***, *p* < 0.001.

Supplementary Figure 4 Flow cytometry gating strategy for the analysis of antigen-specific T cell responses and Tfh-associated CD4^+^ T cell responses. **(A)** Representative gating strategy used to identify cytokine-producing CD4^+^ and CD8^+^ T cells following antigen stimulation. Lymphocytes were first selected based on forward and side scatter (FSC/SSC) parameters, followed by exclusion of doublets using FSC-A versus FSC-H and selection of viable cells using a viability dye. CD3^+^ T cells were then identified and further subdivided into CD4^+^ and CD8^+^ T cell populations. Antigen-specific functional responses were assessed within each subset by intracellular staining for IFNγ, TNFα and IL-2, as well as for surface expression of the degranulation marker CD107a. Representative plots illustrating the gating strategy for CD4^+^ (purple) and CD8^+^ (green) T cell functional analyses are shown. **(B)** Representative gating strategy used to identify Tfh-associated CD4^+^ T cell response. Following selection of singlets and viable lymphocytes, CD4^+^ T cells were gated after exclusion of CD19^+^ B cells. Antigen-specific Tfh-associated responses were defined as CD4^+^ T cells expressing IL-21 and/or IFNγ and/or CD154 (CD40L) and/or IL-4 following antigen stimulation.
