# Supplementary figures and images for: Preclinical evaluation of a multi-epitope mRNA vaccine platform for broad and durable SARS-CoV-2 protection
Source: Front Immunol. 2026 May 5;17:1787877. doi: 10.3389/fimmu.2026.1787877 (PMC13183823; doi:10.3389/fimmu.2026.1787877)

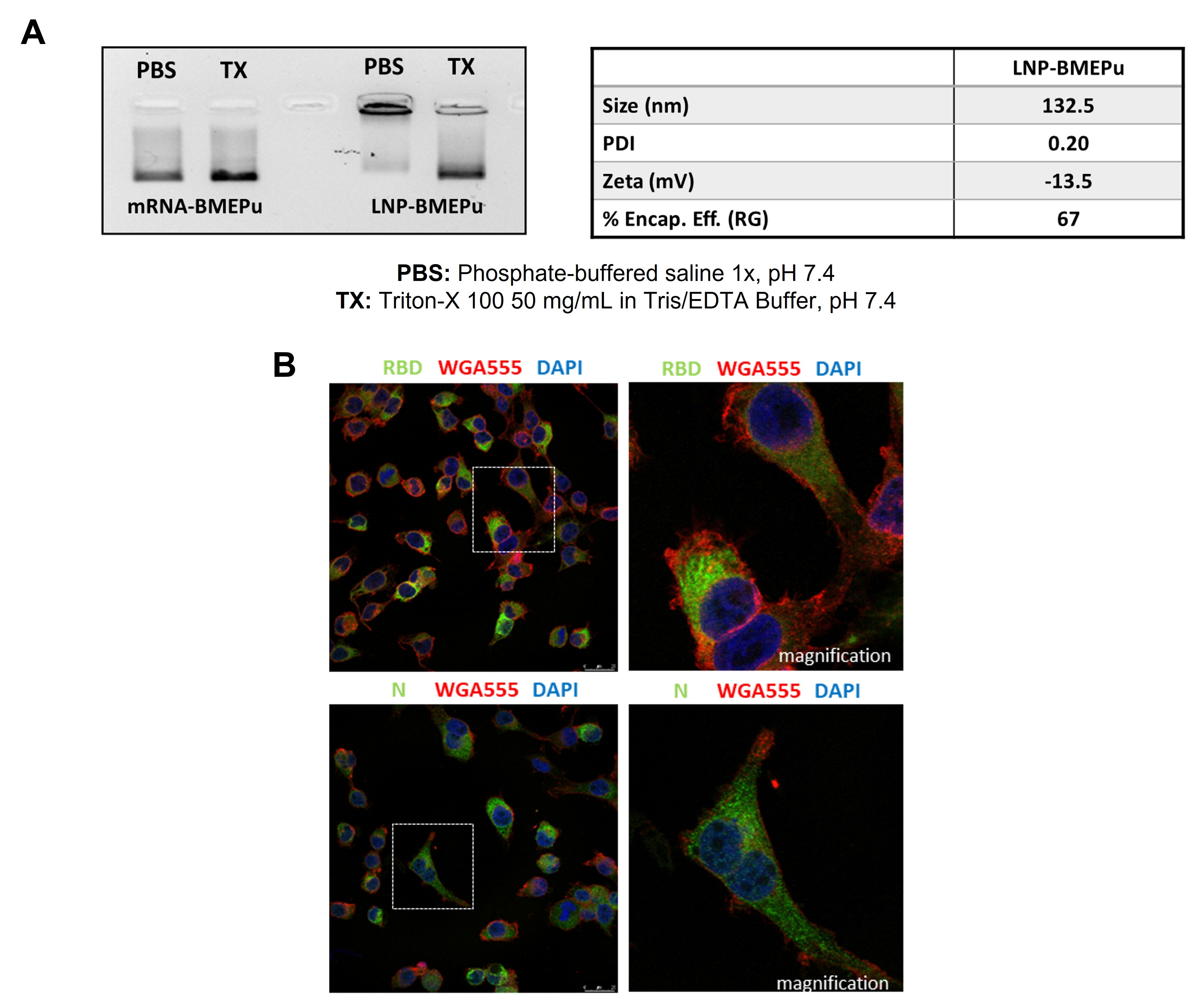

Supplement: Supplementary Figure 1 — In vitro characterization of LNP-BMEPu particle. (A) mRNA concentration and estimated RNA encapsulation efficiency using RiboGreen (RG) and denaturing formaldehyde agarose gel assays (representative; left panel). Measurement of main physicochemical characteristics of LNP-BMEPu particles (right panel). (B) Subcellular distribution of BMEPu protein in HeLa cells transfected with 5 µg of LNP-BMEPu by confocal microscopy. Cells were fixed at 6 h.p.t. and labelled with WGA-555 and anti-RBD or anti-N antibodies (Alexa-488). The images on the right correspond to the magnification of the regions indicated by the discontinuous white square in the images on the left. Cells were stained with DAPI to detect cell nuclei. [file Image1.jpeg]

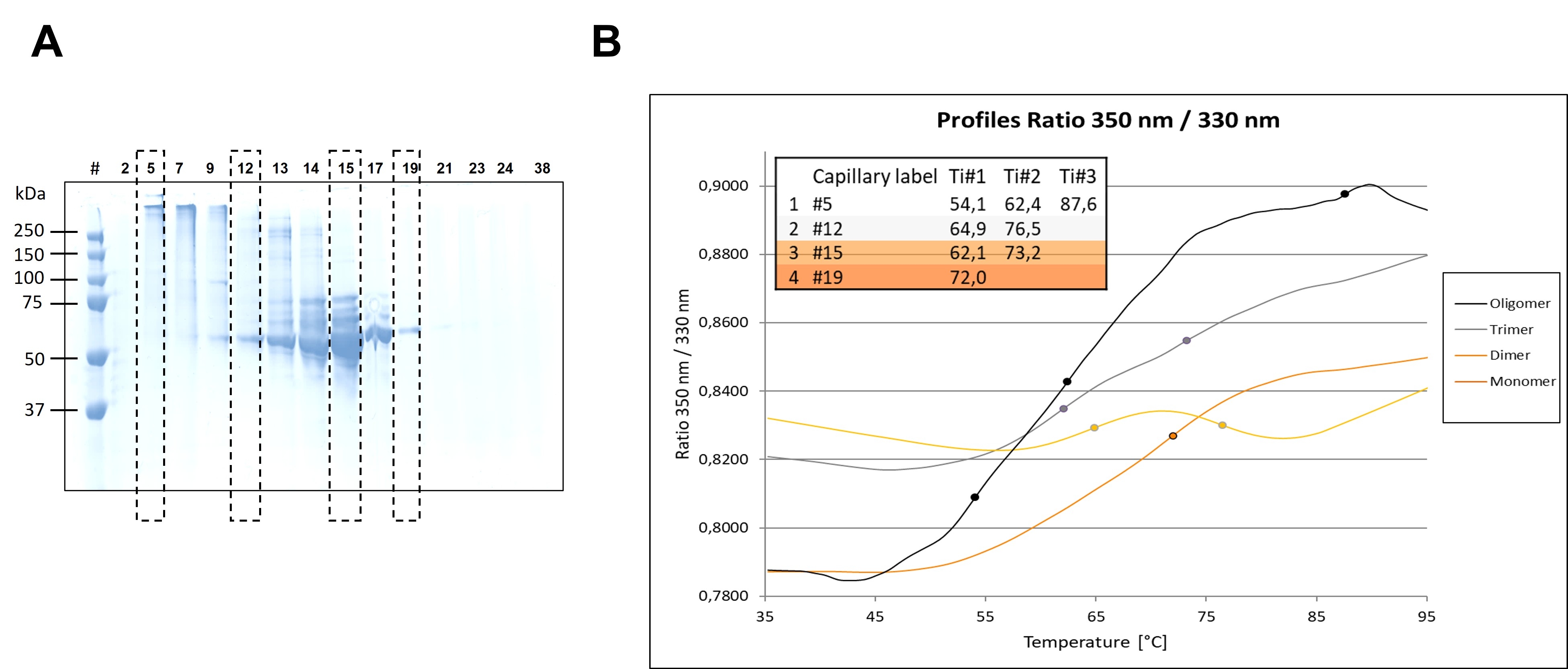

Supplement: Supplementary Figure 2 — Oligomerization analysis of the CoV2-BMEPu protein. (A) Coomassie staining of Native-PAGE analysis of BMEPu under non-denaturing conditions, preserving higher-order oligomeric assemblies. (B) NanoDSF thermal unfolding profile of purified BMEPu protein, showing a cooperative unfolding transition at approximately 87 °C, indicative of high thermal stability in solution. [file Image2.jpeg]

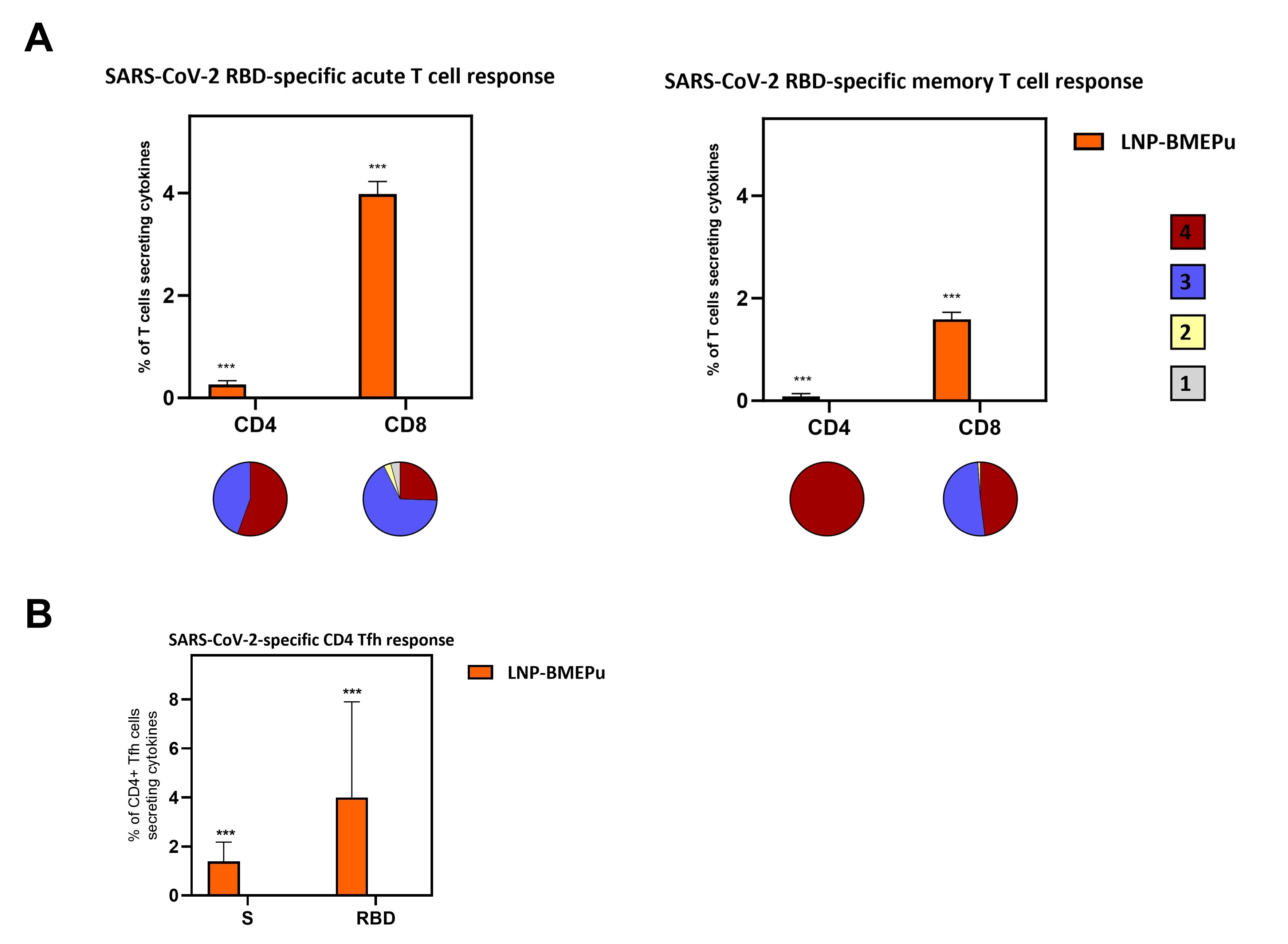

Supplement: Supplementary Figure 3 — (A) Magnitude and polyfunctional profile of the SARS-CoV-2 RBD-specific T cell response. Magnitude of the SARS-CoV-2 RBD-specific acute (left) or memory (right) CD4 or CD8 T cells in the spleens from immunized mice at day 38 and 80, respectively. The overall response represents the sum of the percentages of the SARS-CoV-2 RBD-specific CD4 or CD8 T cells expressing CD107a and/or secreting IFN-γ and/or IL-2 and/or TNF-α. Data are background-subtracted. 95% CI is shown. The polyfunctional profile of the SARS-CoV-2 RBD-specific T cells is represented below each figure. Specific responses are grouped and color-coded based on the number of functions. (B) Magnitude of the SARS-CoV-2-specific Tfh-associated CD4+ T cell responses in spleens from immunized mice at day 38 following stimulation with SARS-CoV-2 S or RBD peptide pools. The total response in each group represents the percentage of Tfh+ cells expressing IL-21 and/or IL-4 and/or IFN-γ and/or CD154 (CD40L). Data are background-subtracted. 95% CI is shown. ***, p < 0.001. [file Image3.jpeg]

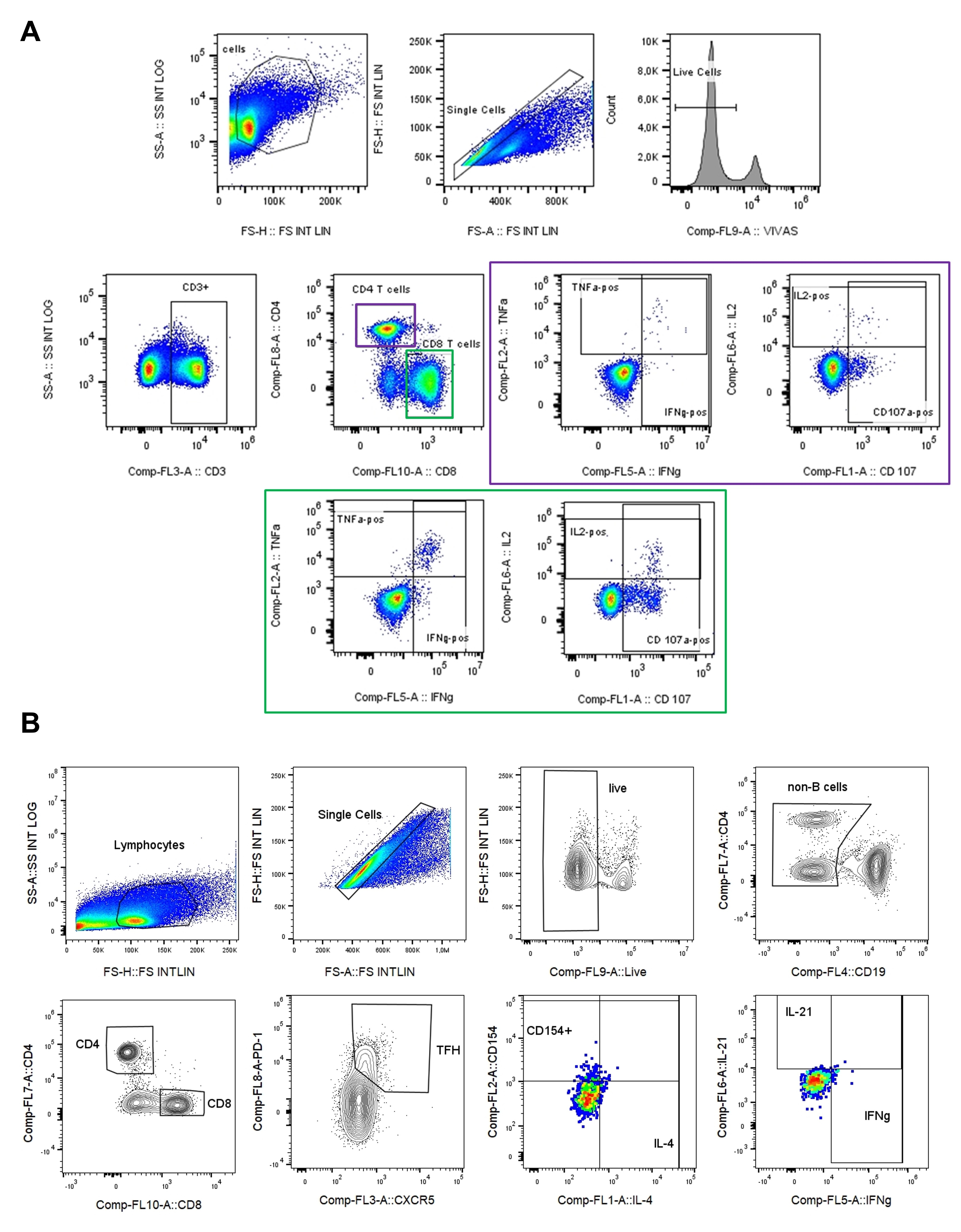

Supplement: Supplementary Figure 4 — Flow cytometry gating strategy for the analysis of antigen-specific T cell responses and Tfh-associated CD4+ T cell responses. (A) Representative gating strategy used to identify cytokine-producing CD4+ and CD8+ T cells following antigen stimulation. Lymphocytes were first selected based on forward and side scatter (FSC/SSC) parameters, followed by exclusion of doublets using FSC-A versus FSC-H and selection of viable cells using a viability dye. CD3+ T cells were then identified and further subdivided into CD4+ and CD8+ T cell populations. Antigen-specific functional responses were assessed within each subset by intracellular staining for IFNγ, TNFα and IL-2, as well as for surface expression of the degranulation marker CD107a. Representative plots illustrating the gating strategy for CD4+ (purple) and CD8+ (green) T cell functional analyses are shown. (B) Representative gating strategy used to identify Tfh-associated CD4+ T cell response. Following selection of singlets and viable lymphocytes, CD4+ T cells were gated after exclusion of CD19+ B cells. Antigen-specific Tfh-associated responses were defined as CD4+ T cells expressing IL-21 and/or IFNγ and/or CD154 (CD40L) and/or IL-4 following antigen stimulation. [file Image4.jpeg]

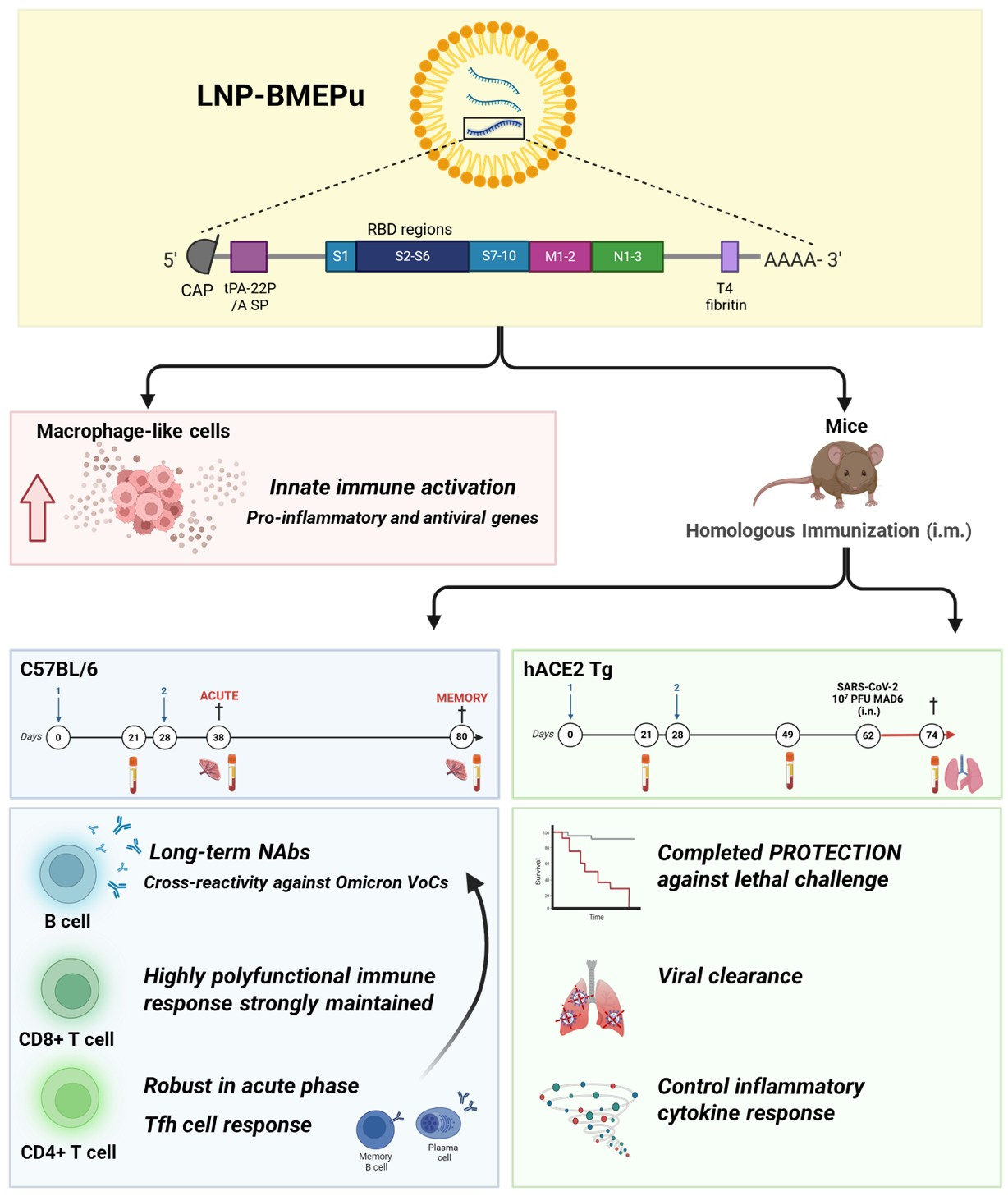

Supplement: Supplementary file 5 [file Image5.tif]
