# Supplementary material for: Preclinical evaluation of a multi-epitope mRNA vaccine platform for broad and durable SARS-CoV-2 protection
Source: Front Immunol. 2026 May 5;17:1787877. doi: 10.3389/fimmu.2026.1787877 (PMC13183823; doi:10.3389/fimmu.2026.1787877)
Supplement: Supplementary file 6 [file Table1.docx]

**Supplementary Table 1.** Predicted MHC Class I Binding Profile of CoV2-BMEPu Patches.

| **Patch ID** | **Strong Binders** (≤0.5% rank) | **Weak Binders** (≤2% rank) | **Best Rank** (%) | **Best IC50** (nM) | **# HLA Alleles** (Strong) | **# HLA Alleles** (Weak) |
| --- | --- | --- | --- | --- | --- | --- |
| S2 | 19 | 46 | 0.02 | 3.7 | 14 | 23 |
| S9 | 17 | 40 | 0.01 | 2.39 | 14 | 23 |
| S1 | 7 | 18 | 0.07 | 48.97 | 6 | 12 |
| S8 | 6 | 16 | 0.04 | 3.66 | 6 | 12 |
| S6 | 3 | 15 | 0.08 | 29.35 | 3 | 10 |
| N1 | 2 | 14 | 0.13 | 76.07 | 2 | 11 |
| S3 | 5 | 13 | 0.18 | 23.59 | 5 | 11 |
| S5 | 5 | 9 | 0.01 | 4.82 | 4 | 7 |
| N3 | 3 | 7 | 0.07 | 32.17 | 3 | 6 |
| M1 | 2 | 6 | 0.15 | 31.09 | 2 | 5 |
| N2 | 4 | 6 | 0.09 | 8.93 | 4 | 6 |
| S4 | 1 | 5 | 0.38 | 135.44 | 1 | 4 |
| S7 | 2 | 5 | 0.21 | 277.45 | 2 | 5 |
| M2 | 0 | 3 | 0.73 | 179.94 | 0 | 3 |
| S10 | 0 | 2 | 1.2 | 779.41 | 0 | 2 |

Binding predictions were performed using NetMHCpan (IEDB default implementation; BA-based predictor) across the IEDB “most frequent HLA-A and HLA-B” reference allele panel. Strong binders were defined as percentile rank ≤0.5%, and weak binders as percentile rank ≤2%, with strong binders constituting a subset of the relaxed-threshold (≤2%) binders. Best rank corresponds to the lowest percentile value predicted for any peptide fragment within each patch. IC50 values are reported for the top-ranking peptide.

**Supplementary Table 2.** Predicted MHC Class II Binding Profile of CoV2-BMEPu Patches.

| **Patch ID** | **Strong Binders** (≤2% rank) | **Weak Binders** (≤10% rank) | **Best Rank** (%) | **Best IC50** (nM) | **# HLA Alleles** (Strong) | **# HLA Alleles** (Weak) | **Representative Core Peptide** |
| --- | --- | --- | --- | --- | --- | --- | --- |
| S9 | 21 | 83 | 0.48 | 6.54 | 7 | 14 | YRFNGIGVT |
| S2 | 13 | 75 | 0.03 | 7.42 | 4 | 11 | FASVYAWNR |
| S3 | 9 | 15 | 0.82 | 39.11 | 2 | 3 | IRGDEVRQI |
| S8 | 9 | 25 | 0.32 | 12.74 | 2 | 8 | LLFNKVTLA |
| S6 | 4 | 33 | 0.08 | 4.03 | 1 | 10 | FELLHAPAT |
| N1 | 4 | 9 | 0.43 | 19.88 | 1 | 2 | IVLQLPQGT |
| N2 | 3 | 6 | 1.1 | 46 | 3 | 6 | LLLDRLNQL |
| S1 | 0 | 1 | 8.8 | 530.23 | 0 | 1 | LHSTQDLFL |
| S10 | 0 | 2 | 3.8 | 179.85 | 0 | 1 | YFKNHTSPD |
| M2 | 0 | 9 | 2.1 | 85.04 | 0 | 4 | YKLNTDHSS |
| N3 | 0 | 14 | 2.8 | 15.8 | 0 | 3 | VTLLPAADL |

Binding predictions were performed using the IEDB-recommended MHC-II binding predictor (BA-based method; default settings) across the IEDB “most frequent 26 HLA-DR” reference allele panel. Strong binders were defined as percentile rank ≤2%, and weak binders as percentile rank ≤10%, with strong binders constituting a subset of the relaxed-threshold (≤10%) binders. Best rank corresponds to the lowest percentile value predicted within each patch. The representative core peptide corresponds to the highest-scoring predicted binding core sequence.

**Supplementary Table 3.** IEDB-Projected Population Coverage for Predicted HLA Class I Binders Derived from CoV2-BMEPu.

| **Region** | **Coverage (%)** | **Average Hit** | **pc90** |
| --- | --- | --- | --- |
| **World (average)** | **89.03** | **8.59** | **3.42** |
| Europe | 99.68 | 12.2 | 6.32 |
| North America | 99.06 | 11.16 | 5.2 |
| West Indies | 98.98 | 11.57 | 5.58 |
| East Asia | 98.18 | 10.55 | 3.77 |
| North Africa | 96.03 | 9.4 | 3.55 |
| West Africa | 95.49 | 10.1 | 3.55 |
| South Asia | 94.73 | 9.15 | 3.69 |
| Oceania | 94.71 | 7.27 | 3.21 |
| Northeast Asia | 94.70 | 8.83 | 3.52 |
| Southeast Asia | 94.56 | 8.52 | 3.34 |
| South Africa | 93.03 | 8.62 | 3.36 |
| Southwest Asia | 92.50 | 8.21 | 3.19 |
| East Africa | 90.78 | 8.21 | 3.05 |
| South America | 88.30 | 6.18 | 1.71 |
| Central Africa | 86.04 | 7.12 | 1.43 |
| Central America | 7.76 | 0.43 | 0.33 |

Population coverage was calculated using the IEDB Population Coverage tool based on BA-derived predicted binders summarized in Supplementary Table 2. Coverage calculations were performed using predicted binders defined at the relaxed percentile threshold (≤2%), which includes strong binders (≤0.5%) as a subset. Coverage represents the projected fraction of individuals predicted to present at least one epitope. Average hit corresponds to the mean number of epitope-HLA combinations recognized per individual, and pc90 indicates the minimum number recognized by 90% of the population.

**Supplementary Table 4.** IEDB-Projected Population Coverage for Predicted HLA Class II Binders Derived from CoV2-BMEPu.

| **Region** | **Coverage (%)** | **Average Hit** | **pc90** |
| --- | --- | --- | --- |
| **World (average)** | **64.55** | **2.93** | **0.7** |
| North America | 87.89 | 4.51 | 1.65 |
| Europe | 85.83 | 4.22 | 1.41 |
| East Asia | 81.82 | 4.4 | 1.1 |
| South Asia | 75.38 | 3.23 | 0.81 |
| North Africa | 75.06 | 3.2 | 0.8 |
| West Indies | 69.22 | 3.29 | 0.65 |
| East Africa | 68.3 | 3.16 | 0.63 |
| West Africa | 65.23 | 3.07 | 0.58 |
| Central Africa | 62.71 | 2.82 | 0.54 |
| Northeast Asia | 59.99 | 2.61 | 0.5 |
| Oceania | 59.87 | 2.7 | 0.5 |
| South America | 58.59 | 2.55 | 0.48 |
| Southeast Asia | 56.98 | 2.39 | 0.46 |
| Central America | 49.91 | 2.06 | 0.4 |
| Southwest Asia | 43.93 | 1.74 | 0.36 |
| South Africa | 32.1 | 0.88 | 0.29 |

Population coverage was calculated using the IEDB Population Coverage tool based on BA-derived predicted binders summarized in Supplementary Table 2. Coverage calculations were performed using predicted binders defined at the relaxed percentile threshold (≤2%), which includes strong binders (≤0.5%) as a subset. Coverage represents the projected fraction of individuals predicted to present at least one epitope. Average hit corresponds to the mean number of epitope-HLA combinations recognized per individual, and pc90 indicates the minimum number recognized by 90% of the population.

**Supplementary Table 5:** TaqMan Probes and Primer Sets Used for Quantification of Host Inflammatory Genes and SARS-CoV-2 RNA.

Mouse **(A)** and Human **(B)** TaqMan probes.

| **A: Gene (Mouse)** | **TaqMan Assay ID** |
| --- | --- |
| *IP10 (CXCL10)* | Mm00445235_m1 |
| *CCL2* | Mm00441242_m1 |
| *CCL12* | Mm01617100_m1 |
| *IFNγ* | Mm00116864_m1 |
| *IL6* | Mm00446190_m1 |
| **B: Gene (Human)** | **TaqMan Assay ID** |
| *TNFα* | Hs00174128_m1 |
| *IL6* | Hs00174131_m1 |
| *IL12β* | Hs01011518_m1 |
| *CXCL8 (IL8)* | Hs00174103_m1 |
| *CXCL10* | Hs00171042_m1 |
| *IFIT1* | Hs03027069_m1 |

**C.** Custom Primer and Probe Sets for Viral RNA and Host ISG Quantification.

| **Target Gene** | **Oligonucleotide** | **Sequence (5′ → 3′)** |
| --- | --- | --- |
| *28S (housekeeping)* | Probe [JOE] | TAGTAGCTGGTTCCCTCCGAAGTTTCCCT |
|  | Forward | GGGCAAAGACTAATCGAACCAT |
|  | Reverse | CGAGAGCAGCTATCCT |
| *RdRp* | Probe [JOE] | AGAGCTATGAATTGCCAGAC |
|  | Forward | GTGARATGGTCATGTGTGGCG |
|  | Reverse | CARATGTAAASACACTATAGC |
| *E* | Probe [JOE] | ACACTAGCCATCCTTACTGCGCTTCGC |
|  | Forward | ACAGGTAGTTAAATAGTTAATAGCGT |
|  | Reverse | ATATTGCAGCAGTACGCACACA |

**D.** Custom Primer Sets for Host ISG Quantification.

| **Gene** | **Primer** | **Sequence (5′ → 3′)** |
| --- | --- | --- |
| *MxA* | Forward | GGGCAAAGACTAATCGAACCAT |
|  | Reverse | CGAGAGCAGCTATCCT |
| *IFIT2* | Forward | CCCAGCAATTCAGGTGTAACA |
|  | Reverse | ACAAGGCCATCCACCACTTAT |
| *IFNγ* | Forward | CGACCTCGAAACAGCATCTG |
|  | Reverse | GAAGGAGATGACTTCGAAAAGC |
| *TRIM22* | Forward | TTGGAAACAGATTTTGGCTTC |
|  | Reverse | GGTTGAGGGGATCGTCAGTA |
| *TRIM5α* | Forward | ATAGATGAGAATCCATGGT |
|  | Reverse | AGGAGTTAAATGTAGTGCT |
| *IL8* | Forward | AGCAGACTAGGGTTGCCAGA |
|  | Reverse | GTAGGGTTGCCAGATGCAAT |
| *IL12* | Forward | AACTGGCGTTGGAAGCACGG |
|  | Reverse | GAACACATGCCCACTTGCTG |
| *CCL2* | Forward | GTCTGGACCCATCCTTCTTGG |
|  | Reverse | GCTACAAGAGGATCACCAGCAG |
| *IFIH1 (MDA5)* | Forward | GCTTCTTGCAAAGTTCTTCC |
|  | Reverse | TTGCCTGGATGTATTACCAC |
| *MIP1α* | Forward | GCAGAGGAGGACAGCAAG |
|  | Reverse | GGTICAGTCCTTTCTTGG |

Commercial TaqMan Gene Expression Assays (Applied Biosystems, Waltham, MA, USA) were used according to the manufacturer’s instructions. Custom primers and probes were synthesized commercially. SARS-CoV-2 genomic *(RdRp*) and subgenomic (*E*) RNA levels were quantified by RT-qPCR as described in Materials and Methods section.
